# Supplementary material for: Engineering intercellular communication using M13 phagemid and CRISPR-based gene regulation for multicellular computing in Escherichia coli
Source: Nat Commun. 2025 Apr 15;16:3569. doi: 10.1038/s41467-025-58760-z (PMC12000618; doi:10.1038/s41467-025-58760-z)
Supplement: Supplementary file 1 — Supplementary Information [file 41467_2025_58760_MOESM1_ESM.pdf]

Supplementary information

Engineering phagemid-based intercellular communication for distributed computing in *Escherichia coli* consortium

Hadiastri Kusumawardhani[1], Florian Zoppi[1], Roberto Avendaño-Vega[1], Yolanda Schaerli[1]

[1]Department of Fundamental Microbiology, Faculty of Biology and Medicine, University of Lausanne, Lausanne, Switzerland

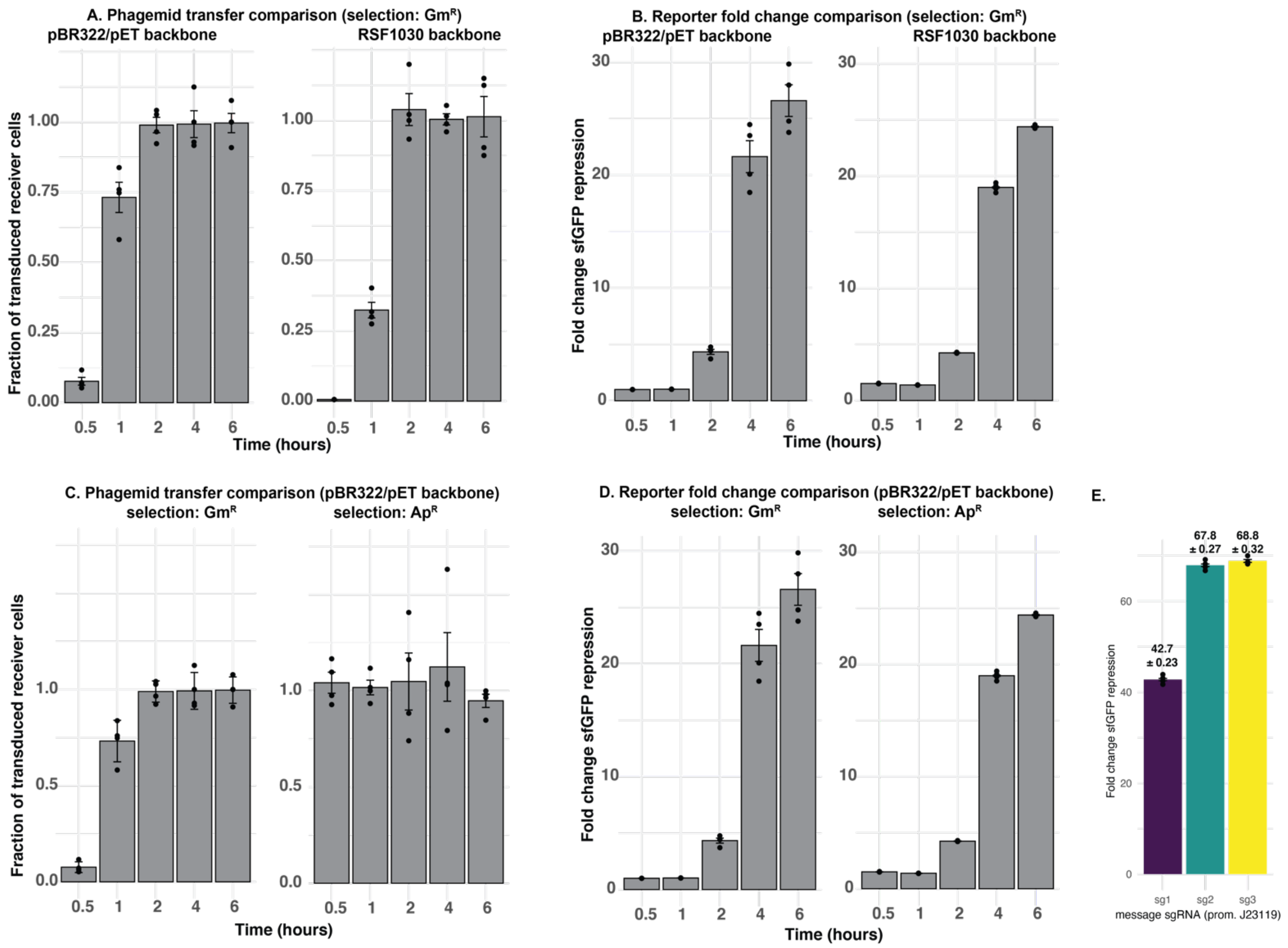

Figure S1. Characterization of phagemid transfer. A. Transduction of the pBR322 phagemid compared to the RSF1030 message phagemid (both with gentamicin resistance) over time. The initial sender to receiver ratio was 2:1. Transduction rates were determined by counting colony-forming units of successfully transduced cells on selective plates divided by the total amount of receiver colonies. **B.** Fold-change of sfGFP reporter repression over time for pBR322 and RSF1030-based phagemids. **C.** Transduction of pBR322-based message phagemid with gentamicin (sender-to-receiver ratio 2:1) or ampicillin resistances (sender-to-receiver 1:1) overtime. **D.** Fold-change of sfGFP reporter repression over time for pBR322-based message phagemid selected by gentamicin resistance or by ampicillin resistance. **E.** Fold-change of sfGFP reporter repression for pBR322-based phagemid with promoter J23119 regulating the expression of sgRNA-1, sgRNA-2, or sgRNA-3. Data in A, B, C, D, and E represent the mean +/- SD of 4 independent biological replicates.

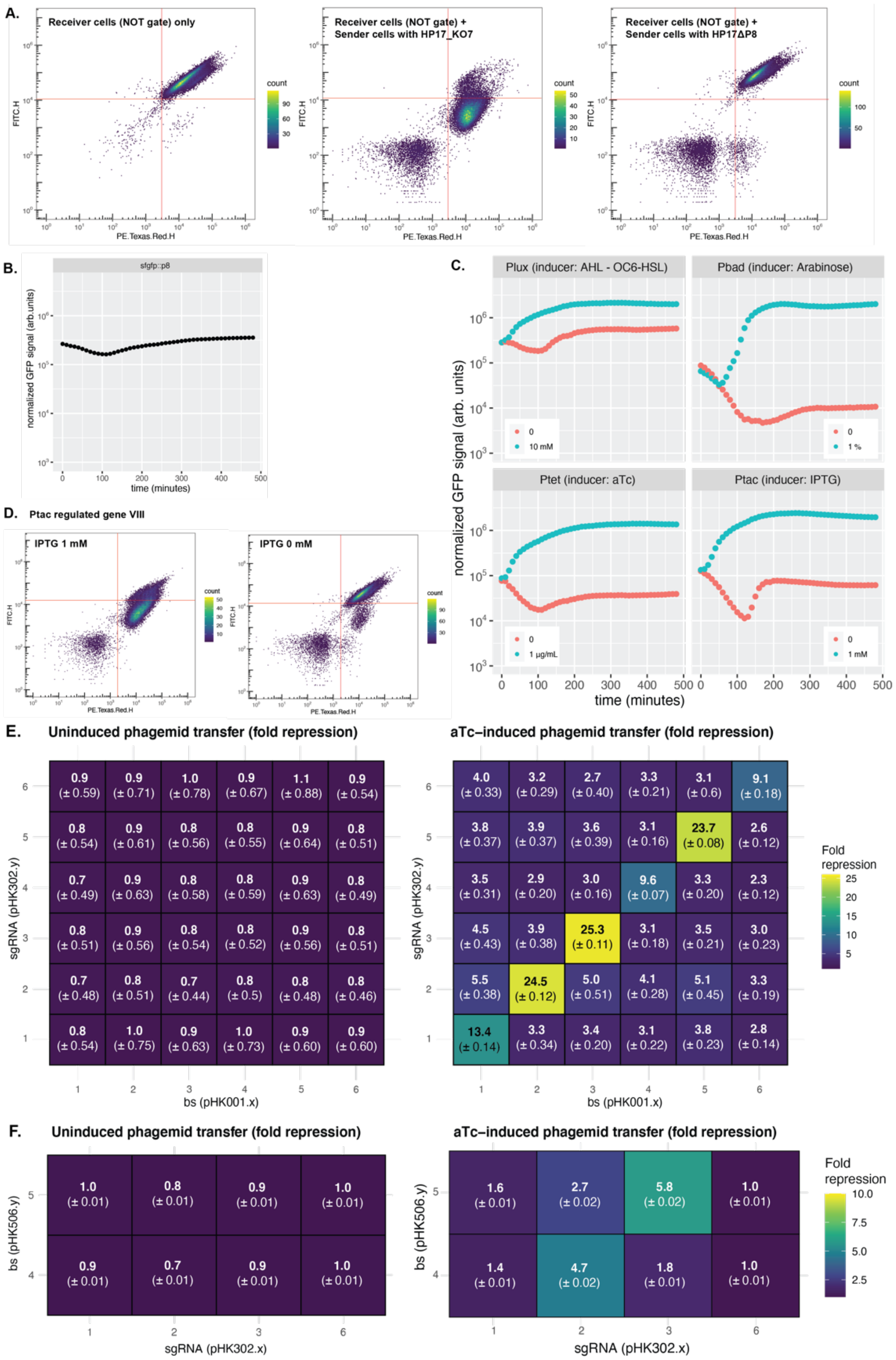

Figure S2. Characterization of inducible phagemid transfer system. A. Gene VIII knock-out prevents phagemid transfer. Flow cytometry measurement of sfGFP (FITC-H) and mCherry (PE.Texas Red H) reporters of NOT gate receiver cells only (left), upon co-culturing of sender cells harboring the complete helper plasmid (HP17-KO) and NOT gate receiver cells (middle) or upon co-culturing sender cells harboring the helper plasmid missing gene VIII (HP17-KOΔP8) and NOT gate receiver cells at 37 °C for 4 hours. **B.** Green fluorescence of sender cells carrying the helper phage HP17_KO7 sfGFP::P8. This plasmid contains a sfGFP reporter gene inserted at the gene VIII locus. **C.** Green fluorescence of the sfGFP reporter gene under the regulation of different inducible promoters (Plux, Pbad, Ptet, Ptac,) on a pCDF plasmid backbone in the absence (red) or presence (blue) of the indicated inducer (AHL, arabinose, aTc, IPTG). **D.** Phagemid transfer of pHK002.2 (pBR322 backbone with sgRNA-2 message) with sender cells carrying the HP17ΔP8 helper plasmid and the second helper plasmid with Ptac upstream of gene VIII in the presence (1 mM) or absence of IPTG. Receiver cells contain the NOT gate plasmid. In the absence of IPTG, a small amount phagemid transfer still occurs demonstrating that Ptac regulation of gene VIII expression is leaky. **E.** Orthogonality assay of aTc-inducible phagemid transfer for NOT gates with different sgRNAs and binding sites pairs in the presence and absence of aTc. **F.** Orthogonality assay of aTc-inducible phagemid transfer for BUF / 'Yes' gates with different sgRNAs and binding sites pairs in the presence and absence of aTc. In this receiver cells's circuit, we placed the sfGFP reporter gene downstream of a promoter J23100 and a binding site for sgRNA-4 (pHK506.4) or sgRNA-5 (pHK506.5). The production of sgRNA-4 (pHK506.4) or sgRNA-5 (pHK506.5) can in turn be repressed by message phagemids containing sgRNA-2 (pHK302.2) or sgRNA-3 (pHK302.3), respectively. Data in E and F represent the mean +/- SD of 3 independent biological replicates. Color codes and numbers in the squares indicate mean+/- SD fold-repression.

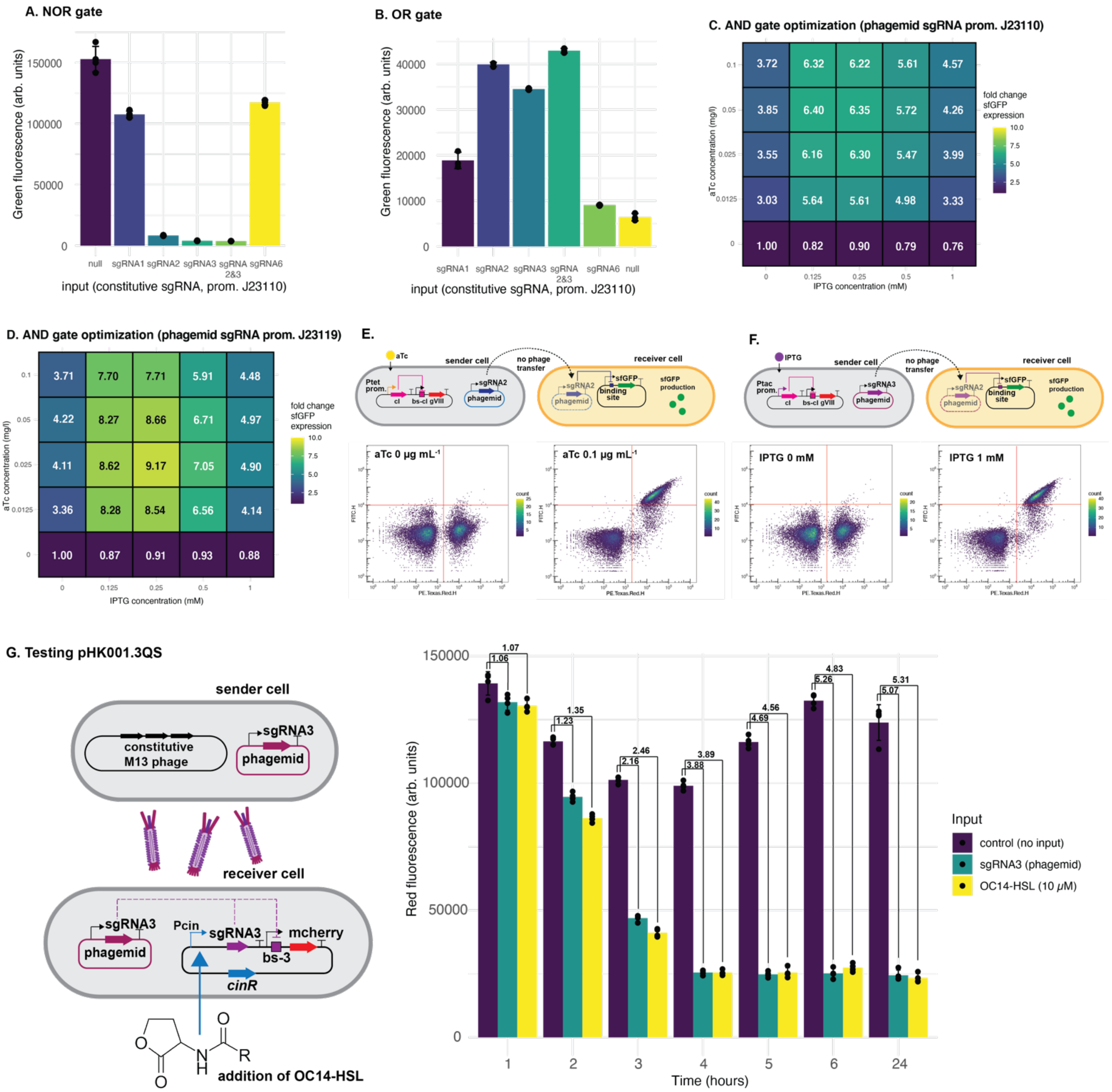

Figure S3. Characterization of two-input logic gates. A. Orthogonality of two-input NOR gate. Receiver cells are co-cultured with sender cells constitutively sending indicated sgRNAs. As expected, sgRNA 2 and 3 repress the fluorescence of the sfGFP reporter, but not sgRNA-1 and sgRNA-6. Data represent the mean +/- SD of 4 independent biological replicates. **B.** Orthogonality of two-input OR gate. Receiver cells are co-cultured with sender cells constitutively sending indicated sgRNAs. sgRNA 2 and 3 lead to stronger fluorescence of the sfGFP reporter than sgRNA-1 and sgRNA-6. Data represent the mean +/- SD of 3 independent biological replicates. **C.** Optimization of two-input AND gates. Promoter J23110 is upstream of the sgRNAs in the phagemid. Different concentrations of arabinose and IPTG were tested. Color codes and numbers in the squares indicate mean fold -change in sfGFP expression. **D.** Optimization of two-input AND gate. Promoter J23119 is upstream of the sgRNAs in the phagemid. Different concentrations of arabinose and IPTG were tested. **E.** Design and characterization of inverter sender cells carrying plasmid pHK-316. Ptet controls expression of the cI gene. Phagemid transfer is occurring in the absence of aTc but is blocked in the presence of aTc. Color codes and numbers in the squares indicate mean fold-change in sfGFP expression. **F.** Design and characterization of inverter sender cells carrying plasmid pHK-326. Ptac controls expression of the cI gene. Phagemid transfer is occurring in the absence of IPTG but is blocked in the presence of IPTG. **G.** Testing the performance of pHK001.3QS by constitutive sending the phagemid (carrying sgRNA3) or direct induction with OC14-HSL (10 µM). Constitutive phagemid sending and direct induction with OC14-HSL elicit similar level of mCherry reporter repression. Data represent the mean +/- SD of 4 independent biological replicates. Fold-changes are indicated above the bar graphs.

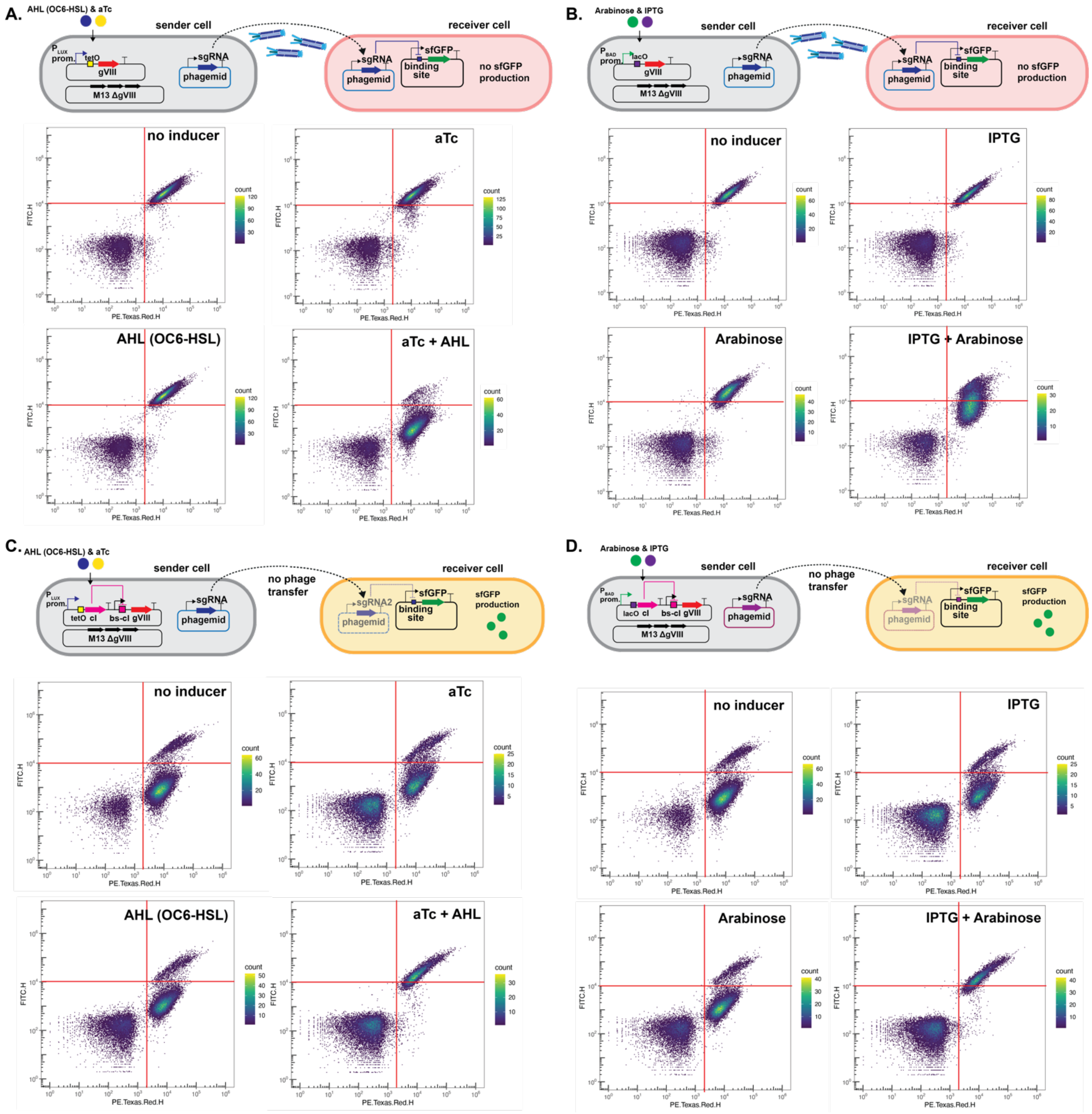

Figure S4. Design and characterization of sender cells for four-input logic gates. A. P_{Lux}-tetO hybrid promoter regulating gene VIII on the second helper plasmid in the sender cells, in the absence or presence of aTc (0.1 mg $liter^{-1}$) and AHL/OC6-HSL (1 mM). **B.** P_{BAD}-lacO hybrid promoter regulating gene VIII on the second helper plasmid in the sender cells, in the absence or presence of IPTG (1 mM) and arabinose (1 %). **C.** P_{Lux}-tetO hybrid promoter regulating cI gene (which represses gene VIII expression) in the presence and absence of aTc (0.1 mg $liter^{-1}$) and AHL/OC6-HSL (1 mM). **D.** P_{BAD}-lacO hybrid promoter regulating cI gene (which represses gene VIII expression) on the second helper plasmid in the sender cells, in the absence or presence of IPTG (1 mM) and arabinose (1 %).

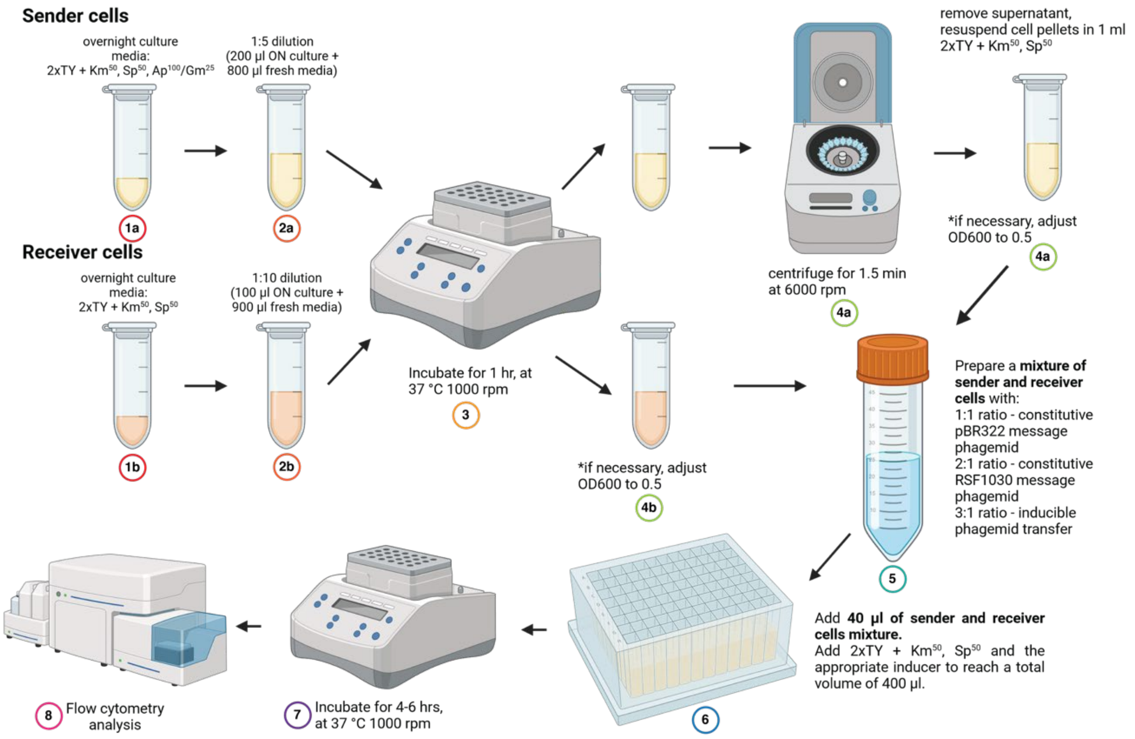

Figure S5. Phagemid transfer assay. 1. We inoculated both sender and receiver cells from single colonies (at least three biological replicates for each sample) and grew them overnight at 37 °C with 1000 rpm shaking in 400 µL of 2x Yeast Extract Tryptone medium (2x YT) with appropriate antibiotics added. **2.** We diluted these overnight cultures ten-fold for receiver cells and five-fold for sender cells into fresh 2x YT media (1 mL). **3.** We grew the cultures at 37 °C with 1000 rpm shaking for 1 hour (early log phase). **4.** Afterwards, we measured ODs of the receiver cell cultures and adjusted them to an OD600 ~ 0.5. For sender cells, we pelleted the cells with centrifugation and removed the supernatant. We resuspended the cell pellets with 2x YT containing kanamycin 50 mg liter^{-1} (for constitutive phagemid transfer) or 2x YT containing kanamycin 50 mg liter^{-1} and spectinomycin 50 mg liter^{-1} (for inducible phagemid transfer) and adjusted samples to an OD600 ~ 0.5. **5.** We mixed sender and receiver cells at 1:1 or 2:1 ratio for experiments with constitutive phagemid production and at a ratio of 3:1 for inducible phagemid production. **6.** We added 40 µL of this mixture, chemical inducer(s) (as indicated), and 360 µL of 2x YT with kanamycin 50 mg liter^{-1} (for constitutive phagemid transfer) or 2x YT with kanamycin 50 mg liter^{-1} and spectinomycin 50 mg liter^{-1} (for inducible phagemid transfer) into a 2 mL 96x deep well plate. **7.** We incubated the deep-well plate at 37 °C with 1000 rpm shaking for 4-6 hours. **8.** Following the incubation, we diluted the samples 200 times with 1x PBS (pH 7.4) and analysed them using a Novocyte Flow cytometer. 'Created in BioRender. Schaerli, Y. (2024) BioRender.com/t68r309

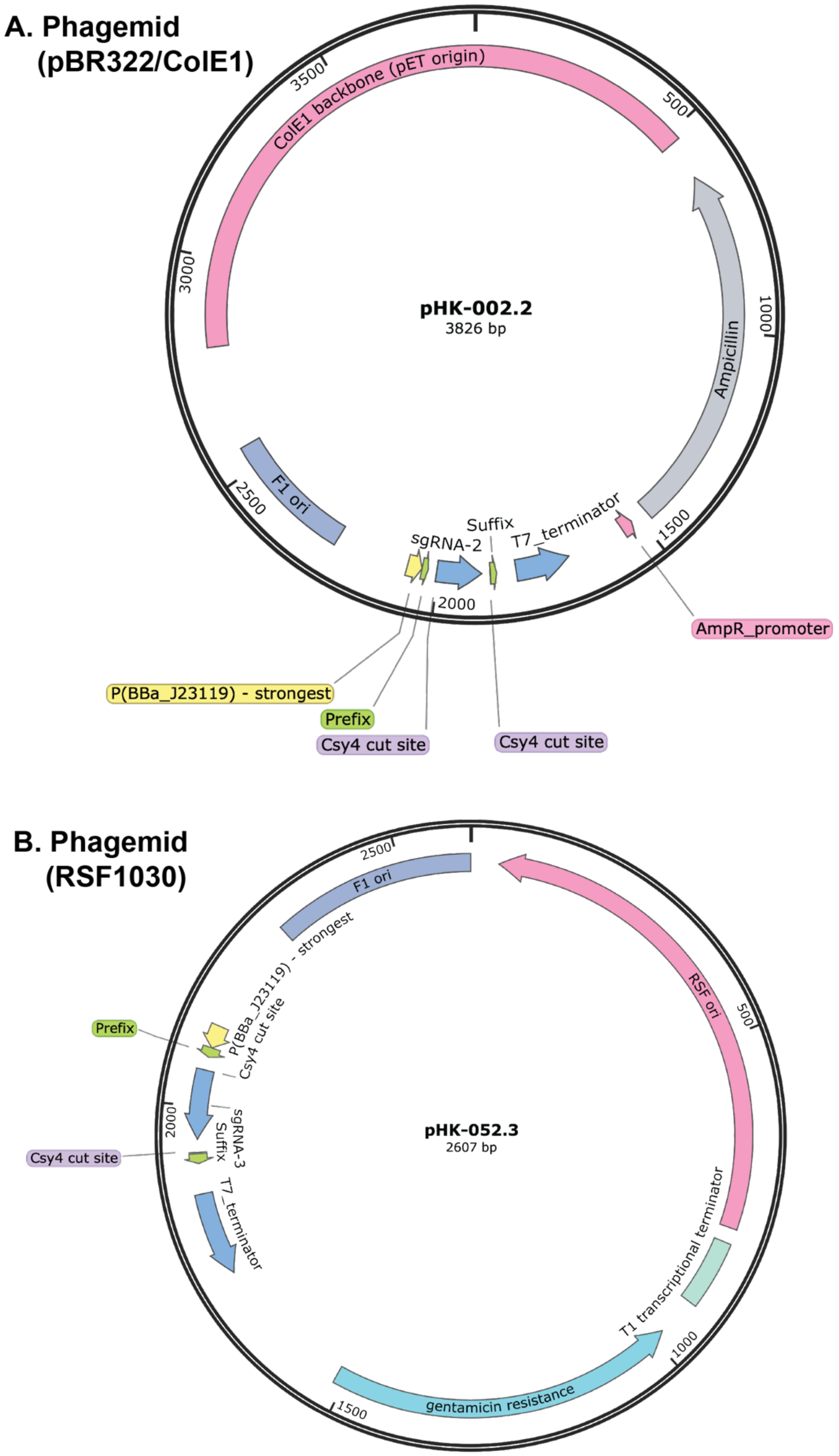

Figure S6. Plasmid maps of message phagemids. A. pBR322-based message phagemid carrying sgRNA-1 under the regulation of J23119 promoter. **B.** RSF1030-based message phagemid carrying sgRNA-2 under the regulation of J23119 promoter. sgRNAs and promoter maybe changed to other variants as listed in Table S2.

A. NOT gate

B. BUF/'Yes' gate

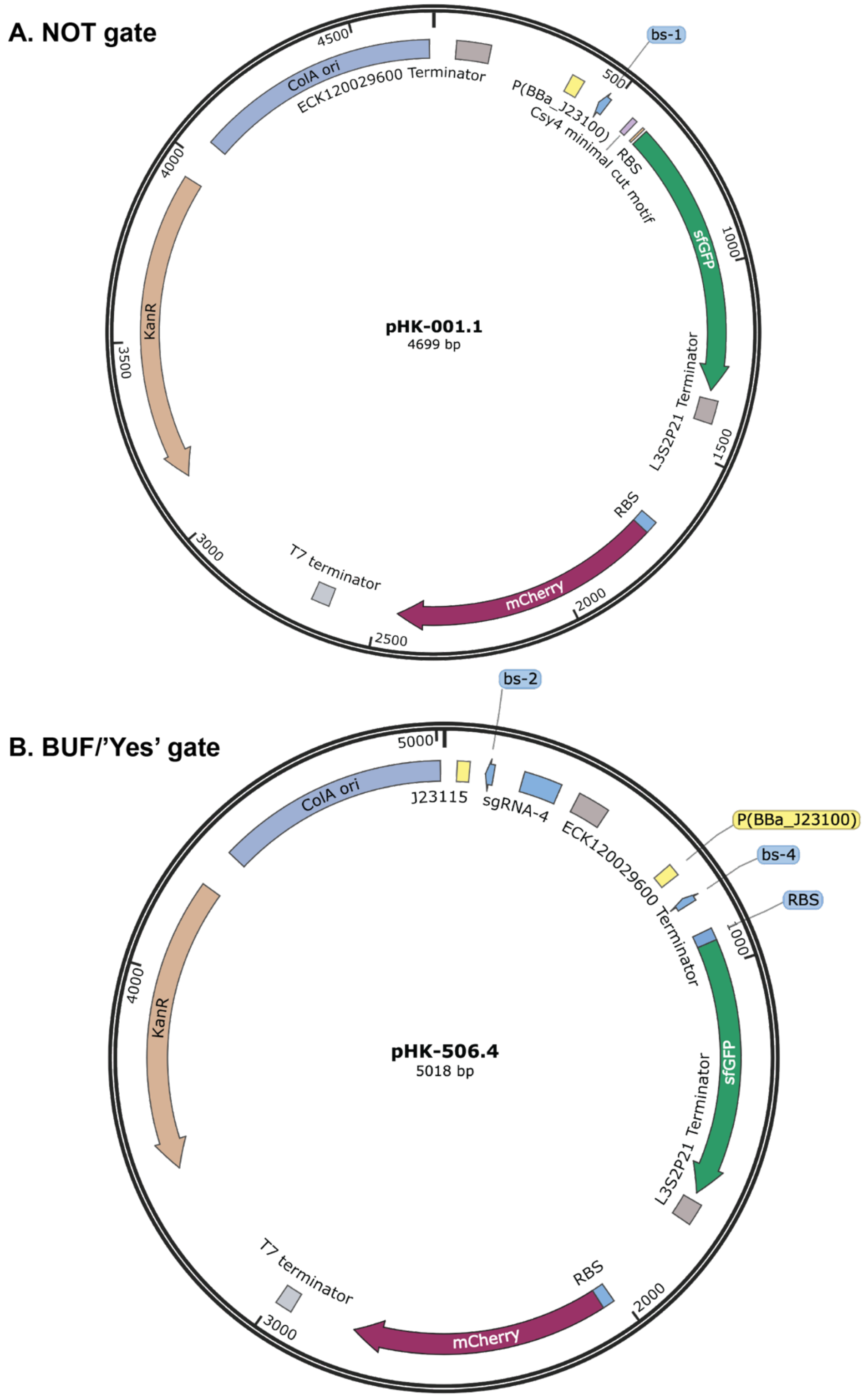

Figure S7. Plasmid maps of one-input logic gate reporter plasmids carried by the receiver cells. A. NOT gate. **B.** BUF/'Yes' gate. sgRNAs and binding sites maybe changed to other variants as listed in Table S2.

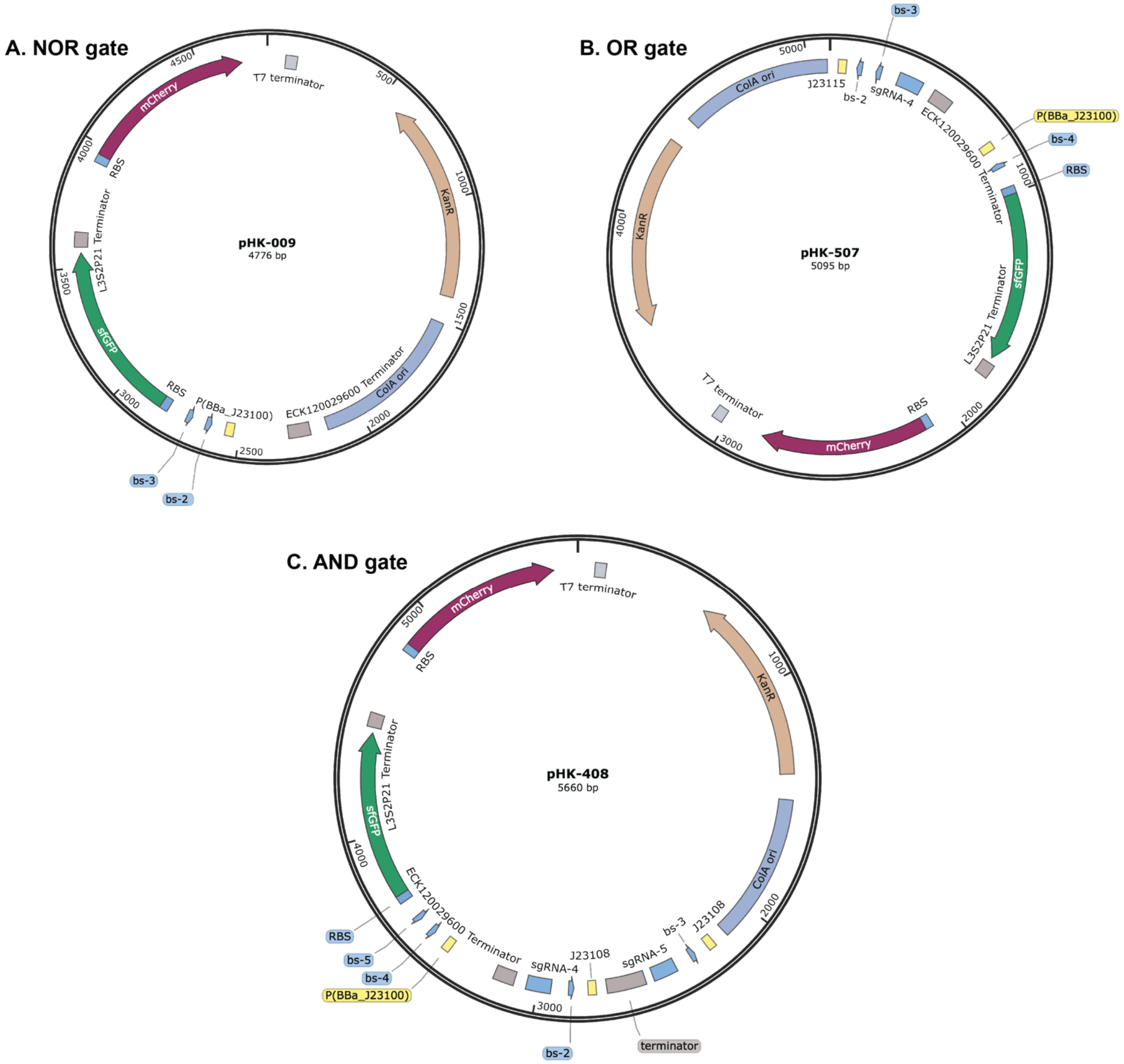

Figure S8. Plasmid maps of two-input logic gate reporter plasmids carried by the receiver cells. A. NOR gate, **B.** OR gate, and **C.** AND gate.

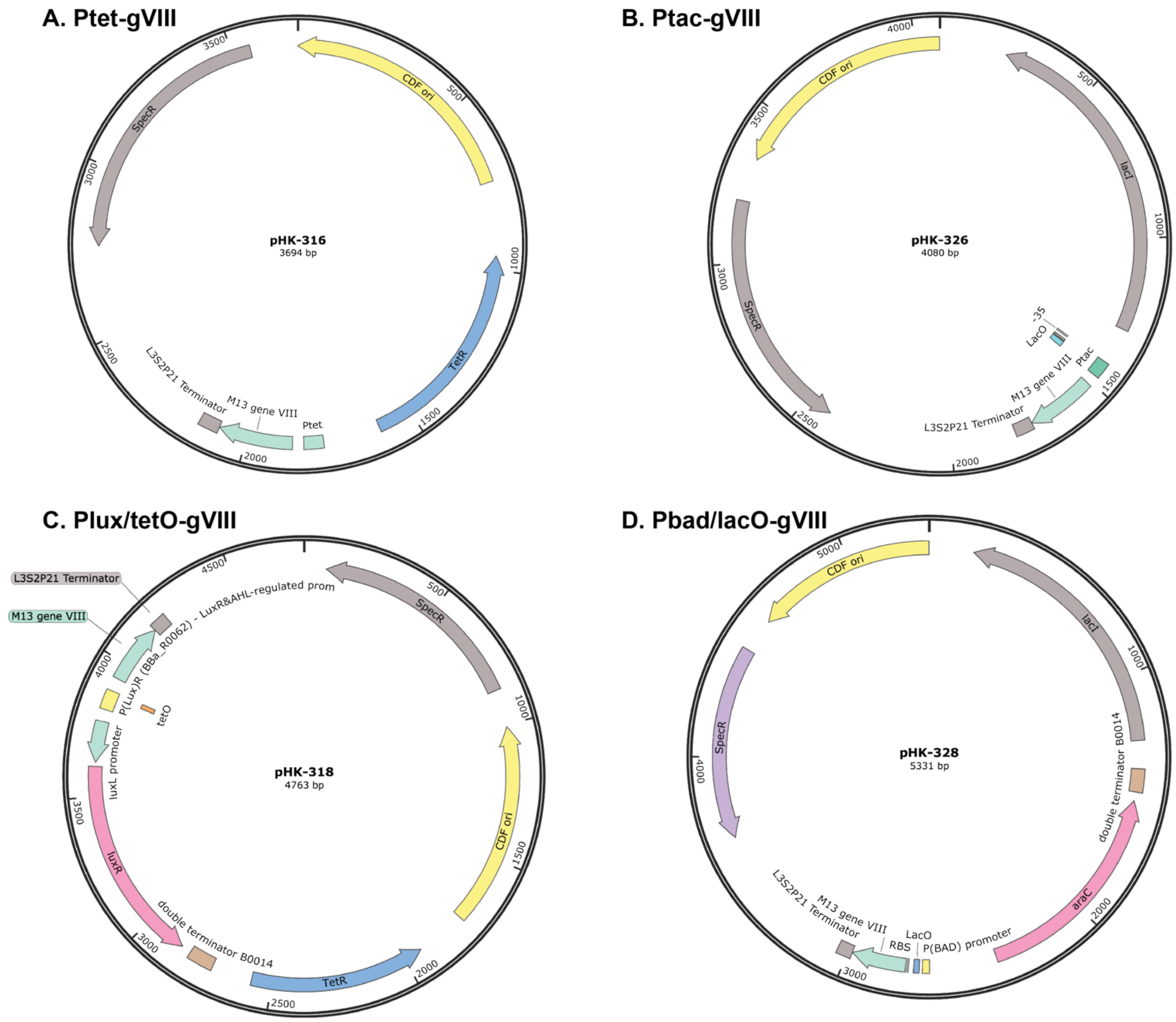

Figure S9. Plasmid maps of the second helper phagemids (pCDF-based), containing inducible gene VIII. A. TetR and Ptet promoter regulating gene VIII expression **B.** LacI and Ptac promoter regulating gene VIII expression. Regulatory proteins and promoters from A and B maybe changed to other variants as listed on Table S2. **C.** TetR, LuxR and Plux-tetO hybrid promoter regulating gene VIII expression **D.** LacI, AraC and Pbad-lacO hybrid promoter regulating gene VIII expression.

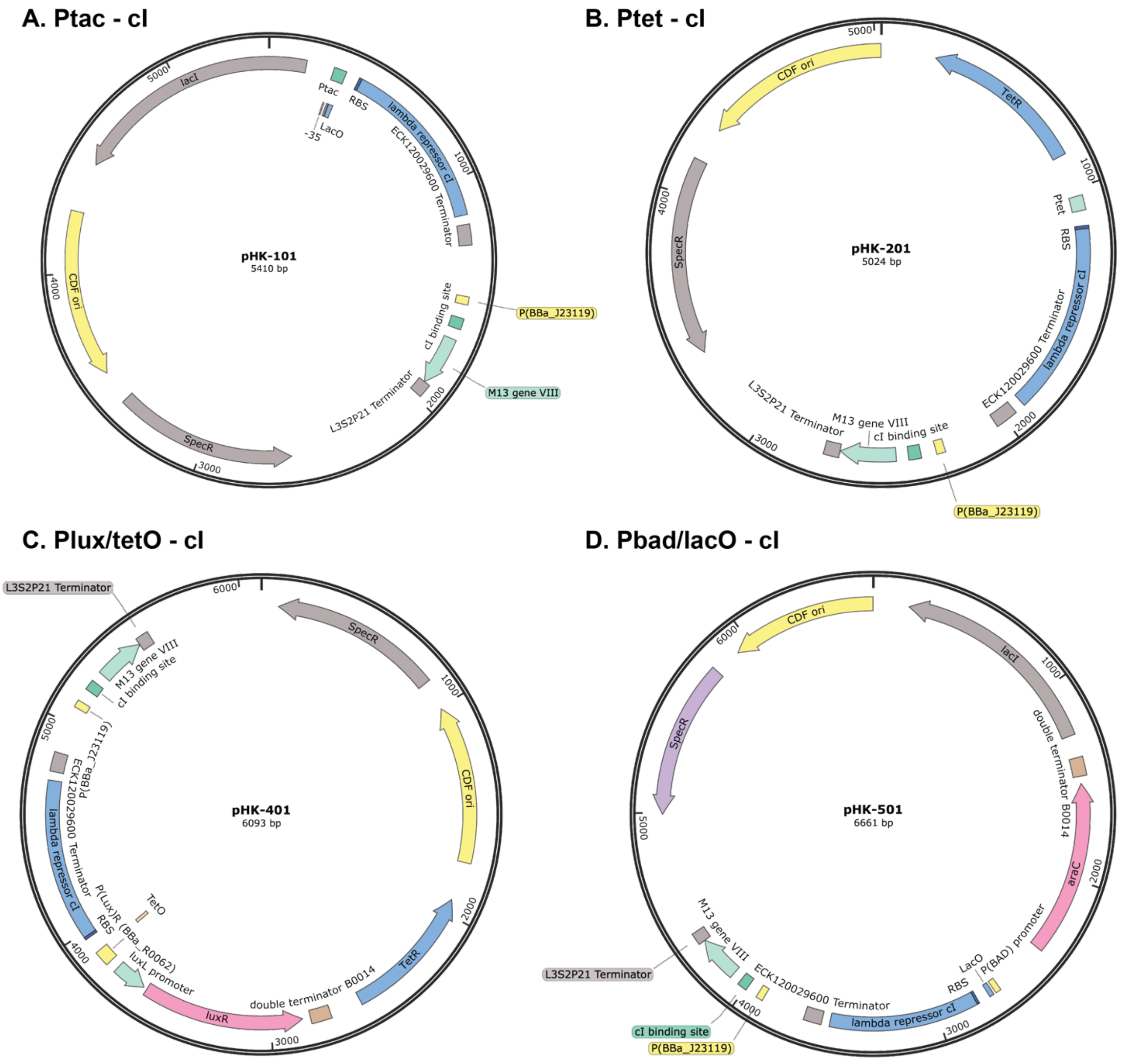

Figure S10. Plasmid maps of the second helper phagemids (pCDF-based), containing inducible lambda repression cI which in turn represses gene VIII expression. A. TetR and Ptet promoter regulating cI expression **B.** LacI and Ptac promoter regulating cI expression. **C.** TetR, LuxR and Plux-tetO hybrid promoter regulating cI expression **D.** LacI, AraC and Pbad-lacO hybrid promoter regulating cI expression.

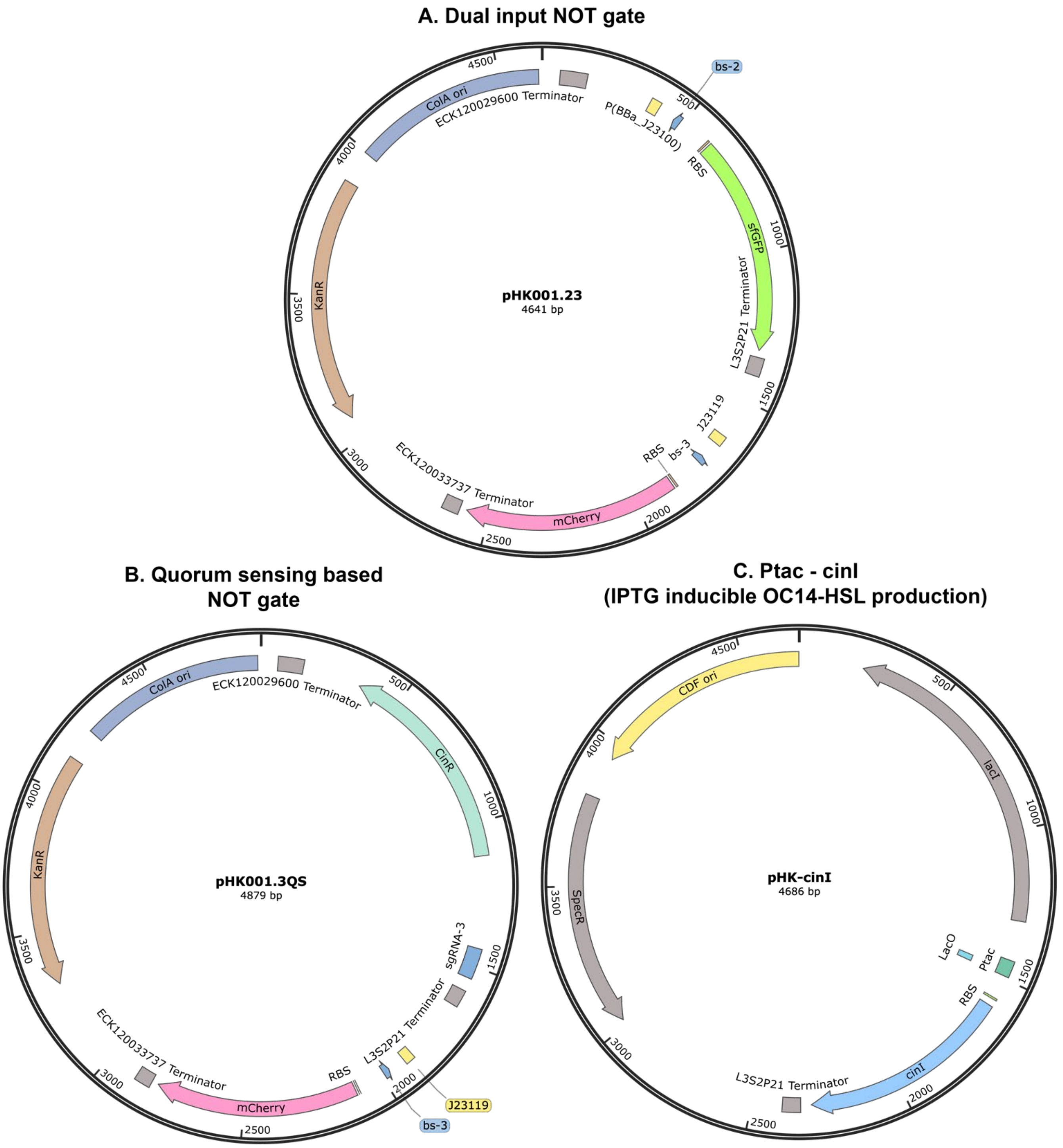

Figure S11. Plasmid maps of A. The dual-input NOT gate **B.** Quorum sensing-based NOT gate **C.** Ptac regulated OC14-HSL (quorum sensing molecule) production.

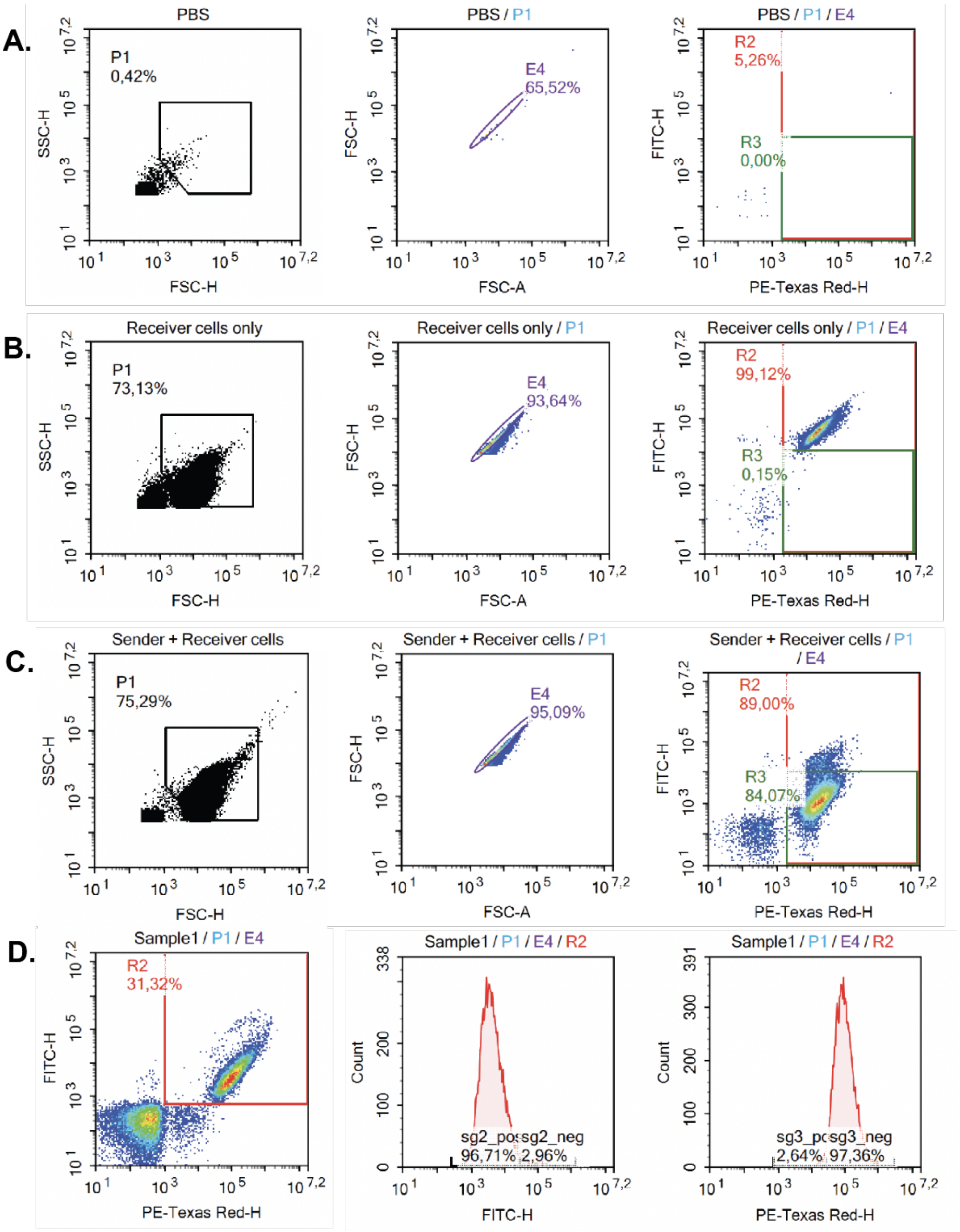

Figure S12. Gating strategy for flow cytometry data analysis. A. Example of Phosphate Buffer Saline (PBS) event measurement, **B.** Example of receiver cells (containing NOT gate) event measurement, **C.** Example of sender and receiver cells (containing NOT gate) event measurement, and **D.** Example of gating used for quantification of sender cells in **Figure 2G.** Briefly, following the phagemid transfer assay, we diluted the samples 200 times with 1x PBS (pH 7.4) and analysed them using a Novocyte Flow cytometer. We used 488 nm excitation laser in combination with FITC filter (emission 530 nm) for sfGFP measurements and 561 nm excitation laser in combination with PE Texas Red filter (emission 615 nm) for mCherry measurements. Flow cytometry raw data were recorded using NovoExpress software (version 1.6.2). First, to discriminate between cells and other particles, all measured events were gated by forward scatter height (FSC.H) > 1000 arbitrary unit (a.u.) and side scatter height (SSC.H) > 200 a.u **(gate P1)**. Second, we excluded doublets by plotting the FSC.H against the forward scatter area (FSC.A) and set a gate for the events with approximately 1:1 ratio of FSC.H to FSC.A **(gate E4)**. We recorded 30,000 events of singlet cells **(gate E4)**. We then set a gate to select for the receiver cells ('sender/receiver threshold' in Figure 2C) for red fluorescence (PE.Texas.Red.H) above 2000 a.u **(gate R2)**. For calculating the fraction of receiver cell population that received the phagemid messages (Figure 2D), we set additional gates for the 'on' state and 'off' sates by setting a threshold for green fluorescence (FITC.H) above or below 10,000 a.u. **(gate R3)**, respectively. For sender cells quantification **(Figure 2G)**, after gating the events with **gate P1** and **gate E4**, we then set a gate to select for the receiver cells by setting thresholds for red fluorescence (PE.Texas.Red.H) above 1000 a.u and green fluorescence (FITC.H) above 1000 a.u. **(gate R2)**. After that, we defined the threshold for receiver cells that received sgRNA-2 **(sg2_pos)** to be below 30,000 a.u at the green fluorescence (FITC.H) channel, otherwise it is classified as not receiving sgRNA-2 **(sg2_neg)**. Similarly, we defined the threshold for receiver cells that received sgRNA-3 **(sg3_pos)** to be below 30,000 a.u at the red fluorescence (PE.Texas.Red.H) channel, otherwise it is classified as not receiving sgRNA-3 **(sg3_neg)**. Cell counts were recorded and fraction of receiver cells with sfGFP repression were reported by dividing **sg2_pos** cell counts from the total of **sg2_pos** and **sg2_neg** cell counts.

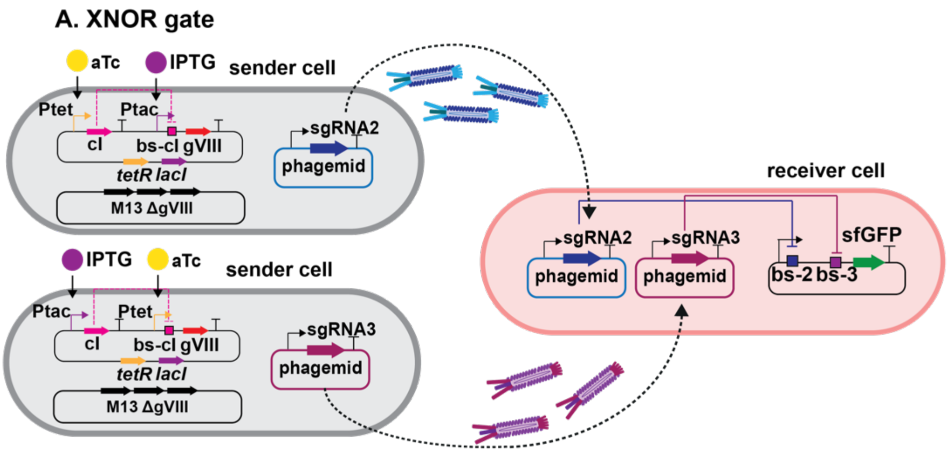

Input 1 IPTG	Input 2 aTc	Output sfGFP
0	0	1
1	0	0
0	1	0
1	1	1

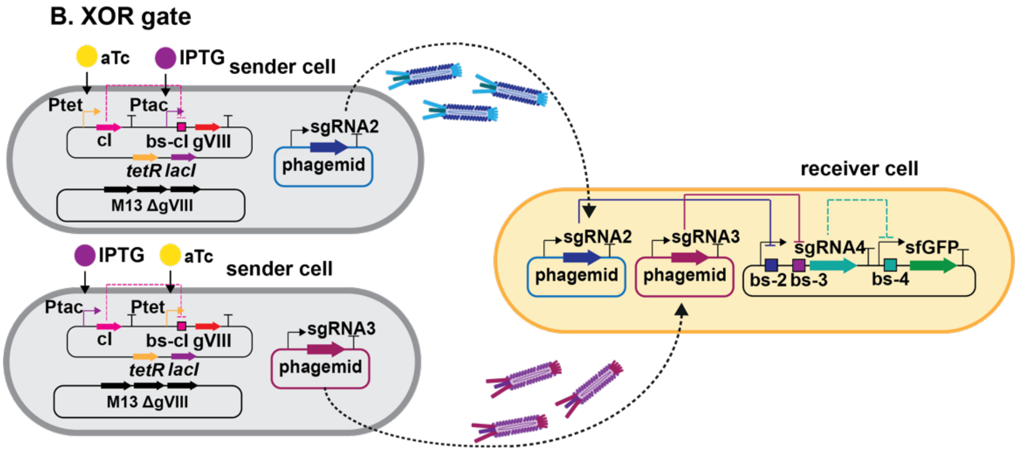

Input 1 IPTG	Input 2 aTc	Output sfGFP
0	0	0
1	0	1
0	1	1
1	1	0

Figure S13. Proposed design of XOR and XNOR gates. The sender cells require only minimal modifications compared to those in the four-input NAND gate. The receiver cells are identical to those used in the two-input OR gate (pHK507) for the XOR gate and the NOR gate (pHK009) for the XNOR gate. Specifically, in the sender cells inducers control cI and gene VIII expression, where cI represses gene VIII. In a sender cell carrying sgRNA2, gene VIII expression is inducible by IPTG via the Ptac promoter, while cI expression is induced by aTc via the Ptet promoter. As a result, IPTG alone triggers phagemid production carrying sgRNA2, whereas the presence of both IPTG and aTc inhibits phage particle formation due to cI-mediated repression of gene VIII. A similar but inverse regulatory mechanism applies to the sender cell carrying sgRNA3. Here, aTc induces gene VIII expression and the corresponding phagemid, while the simultaneous presence of IPTG and aTc inhibits phage production. Thus, when the two sender cells are co-cultured with receiver cells, phagemid particles are produced only when either IPTG or aTc is present, but not when both are added simultaneously.

Table S1. Strains used in this study

Strain name	Description	Strain type	Logic gate	Ref.
DH5α	F⁻ endA1 glnV44 thi-1 recA1 relA1 gyrA96 deoR nupG purB20 φ80dlacZΔM15 Δ(lacZYA-argF)U169, hsdR17(rK⁻mK⁺), λ	cloning	-	41
JM101	glnV44 thi-1 Δ(lac-proAB) F'[lacIqZΔM15 traD36 proAB+]	host	-	29, 30
JM101 Δ*araBAD*	glnV44 thi-1 Δ(lac-proAB) F'[lacIqZΔM15 traD36 proAB+] Δ*araBAD*	host	-	This paper
NOT 001.1	JM101 pHK001.1 + pJ1996v2	Receiver	NOT	This paper
NOT 001.2	JM101 pHK001.2 + pJ1996v2	Receiver	NOT	This paper
NOT 001.3	JM101 pHK001.3 + pJ1996v2	Receiver	NOT	This paper
NOT 001.4	JM101 pHK001.4 + pJ1996v2	Receiver	NOT	This paper
NOT 001.5	JM101 pHK001.5 + pJ1996v2	Receiver	NOT	This paper
NOT 001.6	JM101 pHK001.6 + pJ1996v2	Receiver	NOT	This paper
NOT 001.23	JM101 pHK001.23 + pJ1996v2	Receiver	Dual NOT	This paper
NOT 001.3QS	JM101 pHK001.3QS + pJ1996v2	Receiver	Quorum-sensing NOT	This paper
BUF 506.4	JM101 pHK506.4+ pJ1996v2	Receiver	BUF	This paper
BUF 506.5	JM101 pHK506.5 + pJ1996v2	Receiver	BUF	This paper
OR 507	JM101 pHK507+ pJ1996v2	Receiver	OR, 2- & 4-input NAND	This paper
AND 408	JM101 pHK408+ pJ1996v2	Receiver	AND	This paper
NOR 009	JM101 pHK009 + pJ1996v2	Receiver	NOR	This paper
Constitutive 002.1	JM101 pHK002.1 + HP17_KO7	Sender	NOT	This paper
Constitutive 002.2	JM101 pHK002.2 + HP17_KO7	Sender	NOT	This paper
Constitutive 002.3	JM101 pHK002.3 + HP17_KO7	Sender	NOT	This paper
Constitutive 002.4	JM101 pHK002.4 + HP17_KO7	Sender	NOT	This paper
Constitutive 002.5	JM101 pHK002.5 + HP17_KO7	Sender	NOT	This paper
Constitutive 002.6	JM101 pHK002.6 + HP17_KO7	Sender	NOT	This paper
Constitutive 302.1	JM101 pHK302.1 + HP17_KO7	Sender	NOT	This paper
Constitutive 302.2	JM101 pHK302.2 + HP17_KO7	Sender	NOT	This paper
Constitutive 302.3	JM101 pHK302.3 + HP17_KO7	Sender	NOT	This paper
Constitutive 302.4	JM101 pHK302.4 + HP17_KO7	Sender	NOT	This paper
Constitutive 302.5	JM101 pHK302.5 + HP17_KO7	Sender	NOT	This paper
Constitutive 302.6	JM101 pHK302.6 + HP17_KO7	Sender	NOT	This paper
Inducible Ptet 002.1	JM101 pHK002.1 + HP17ΔP8 + pHK316	Sender	NOT	This paper
Inducible Ptet 002.2	JM101 pHK002.2 + HP17ΔP8 + pHK316	Sender	NOT, 2-input AND	This paper
Inducible Ptet 002.3	JM101 pHK002.3 + HP17ΔP8 + pHK316	Sender	NOT	This paper
Inducible Ptet 302.1	JM101 pHK302.1 + HP17ΔP8 + pHK316	Sender	NOT	This paper
Inducible Ptet 302.2	JM101 pHK302.2 + HP17ΔP8 + pHK316	Sender	NOT, BUF, NOR, OR, 2-input AND	This paper
Inducible Ptet 302.3	JM101 pHK302.3 + HP17ΔP8 + pHK316	Sender	NOT, BUF	This paper
Inducible Ptet 302.4	JM101 pHK302.4 + HP17ΔP8 + pHK316	Sender	NOT	This paper
Inducible Ptet 302.5	JM101 pHK302.5 + HP17ΔP8 + pHK316	Sender	NOT	This paper
Inducible Ptet 302.6	JM101 pHK302.6 + HP17ΔP8 + pHK316	Sender	NOT	This paper
Inducible Ptac 302.2	JM101 pHK302.2 + HP17ΔP8 + pHK326	Sender	NOT	This paper
Inducible Plux 302.2	JM101 pHK302.2 + HP17ΔP8 + pHK336	Sender	NOT	This paper
Inducible Pbad 302.2	JM101 Δ*araBAD* pHK302.2 + HP17ΔP8 + pHK346	Sender	NOT	This paper
Inducible Ptac 052.3	JM101 pHK052.3 + HP17ΔP8 + pHK326	Sender	2-input AND	This paper
Inducible Ptac 352.3	JM101 pHK352.3 + HP17ΔP8 + pHK326	Sender	NOT, BUF, NOR, OR, 2-input AND	This paper
Inducible Plux/tet 002.2	JM101 pHK002.2 + HP17ΔP8 + pHK318	Sender	4-input AND	This paper
Inducible Pbad/lac 052.3	JM101 Δ*araBAD* pHK052.3 + HP17ΔP8 + pHK328	Sender	4-input AND	This paper
Inducible Plux/tet 302.2	JM101 pHK302.2 + HP17ΔP8 + pHK318	Sender	4-input AND	This paper
Inducible Pbad/lac 352.3	JM101 Δ*araBAD* pHK352.3 + HP17ΔP8 + pHK328	Sender	4-input AND	This paper
Inverter Ptet 002.2	JM101 pHK002.2 + HP17ΔP8 + pHK201	Sender	2-input NAND	This paper
Inverter Ptac 052.3	JM101 pHK052.3 + HP17ΔP8 + pHK101	Sender	2-input NAND	This paper
Inverter Plux/tet 002.2	JM101 pHK002.2 + HP17ΔP8 + pHK401	Sender	4-input NAND	This paper
Inverter Pbad/lac 052.3	JM101 Δ*araBAD* pHK052.3 + HP17ΔP8 + pHK501	Sender	4-input NAND	This paper
Inducible OC14-HSL	JM101 pHK-*cinI*	Sender	Quorum sensing NOT	This paper

Table S2. Plasmids used in this study

Plasmid name	Description	Antibiotic resistance	Note	Addgene ID	Ref.
HP17_KO7	pSC101 ori, Kan^R, M13 (gene I-X)	kanamycin	Helper plasmid for constitutive phage transfer; Helper phagemid HP17_KO7 was a gift from Hendrik Dietz	120346	31
HP17ΔP8	pSC101 ori, Kan^R, M13 Δgene VIII	kanamycin	Helper plasmid for regulated phage transfer	235447	This paper
HP17 sfGFP::P8	pSC101 ori, Kan^R, sfGFP::gene VIII	kanamycin	Helper plasmid with sfGFP gene inserted at gene VIII locus	235448	This paper
pJ1996v2	pCDF ori, Spec^R, **node 1:** J23104 - dCas9, **node 3:** J23150 - csy4	spectinomycin	Plasmid encoding for dCas9 & csy4 nuclease for CRISPRi-based circuit	140664	27
pJ1991.2v3	pColA ori, Kan^R, node1: [empty], node2: [empty], node3: [empty]	kanamycin	Backbone plasmid to clone genetic circuits		27
pHK001.1	pColA ori, Kan^R, **node 1:** J23100 – bs1 – sfGFP, **node 3:** J23119 – mCherry	kanamycin	NOT gate receiver with binding site (bs) for sgRNA1	235449	This paper
pHK001.2	pColA ori, Kan^R, **node 1:** J23100 – bs2 – sfGFP, **node 3:** J23119 – mCherry	kanamycin	NOT gate receiver with bs for sgRNA2	235450	This paper
pHK001.3	pColA ori, Kan^R, **node 1:** J23100 – bs3 – sfGFP, **node 3:** J23119 – mCherry	kanamycin	NOT gate receiver with bs for sgRNA3	235451	This paper
pHK001.4	pColA ori, Kan^R, **node 1:** J23100 – bs4 – sfGFP, **node 3:** J23119 – mCherry	kanamycin	NOT gate receiver with bs for sgRNA4	235452	This paper
pHK001.5	pColA ori, Kan^R, **node 1:** J23100 – bs5 – sfGFP, **node 3:** J23119 – mCherry	kanamycin	NOT gate receiver with bs for sgRNA5	235453	This paper
pHK001.6	pColA ori, Kan^R, **node 1:** J23100 – bs6 – sfGFP, **node 3:** J23119 – mCherry	kanamycin	NOT gate receiver with bs for sgRNA6	235454	This paper
pHK506.4	pColA ori, Kan^R, **node 1:** J23115 – bs2 – sgRNA4, **node 2:** J23100 – bs4 – sfGFP, **node 3:** J23119 – mCherry	kanamycin	BUF/YES gate receiver with bs for sgRNA2	235455	This paper
pHK506.5	pColA ori, Kan^R, **node 1:** J23115 – bs2 – sgRNA4, **node 2:** J23100 – bs4 – sfGFP, **node 3:** J23119 – mCherry	kanamycin	BUF/YES gate receiver with bs for sgRNA3	235456	This paper
pHK507	pColA ori, Kan^R, **node 1:** J23115 – bs2 – bs3 – sgRNA4, **node 2:** J23100 – bs4 – sfGFP, **node 3:** J23119 – mCherry	kanamycin	OR/NAND gate receiver with bs for sgRNA2 and sgRNA3	235457	This paper
pHK408	pColA ori, Kan^R, **node 1:** J23115 – bs3 – sgRNA5, **node 2:** J23115 – bs2 – sgRNA4, **node 3:** J23100 – bs4 – bs5 – sfGFP, **node 4:** J23119 – mCherry	kanamycin	AND gate receiver with bs for sgRNA2 and sgRNA3	235458	This paper
pHK009	pColA ori, Kan^R, **node 1:** J23100 – bs2 – bs3 – sfGFP, **node 3:** J23119 – mCherry	kanamycin	NOR gate receiver with bs for sgRNA2 and sgRNA3	235459	This paper
pHK002.1	pBR322 ori, F1 ori, Amp^R, J23119 – sgRNA1	ampicillin	Message phagemid carrying sgRNA1 (prom. J23119, backbone pBR322)	235460	This paper
pHK002.2	pBR322 ori, F1 ori, Amp^R, J23119 – sgRNA2	ampicillin	Message phagemid carrying sgRNA2 (prom. J23119, backbone pBR322)	235461	This paper
pHK002.3	pBR322 ori, F1 ori, Amp^R, J23119 – sgRNA3	ampicillin	Message phagemid carrying sgRNA3 (prom. J23119, backbone pBR322)	235462	This paper
pHK002.4	pBR322 ori, F1 ori, Amp^R, J23119 – sgRNA4	ampicillin	Message phagemid carrying sgRNA4 (prom. J23119, backbone pBR322)	235463	This paper

pHK002.5	pBR322 ori, F1 ori, Amp^R, J23119 – sgRNA5	ampicillin	Message phagemid carrying sgRNA5 (prom. J23119, backbone pBR322)	235464	This paper
pHK002.6	pBR322 ori, F1 ori, Amp^R, J23119 – sgRNA6	ampicillin	Message phagemid carrying sgRNA6 (prom. J23119, backbone pBR322)	235465	This paper
pHK302.1	pBR322 ori, F1 ori, Amp^R, J23110 – sgRNA1	ampicillin	Message phagemid carrying sgRNA1 (prom. J23110, backbone pBR322)	235466	This paper
pHK302.2	pBR322 ori, F1 ori, Amp^R, J23110 – sgRNA2	ampicillin	Message phagemid carrying sgRNA2 (prom. J23110, backbone pBR322)	235467	This paper
pHK302.3	pBR322 ori, F1 ori, Amp^R, J23110 – sgRNA3	ampicillin	Message phagemid carrying sgRNA3 (prom. J23110, backbone pBR322)	235468	This paper
pHK302.4	pBR322 ori, F1 ori, Amp^R, J23110 – sgRNA4	ampicillin	Message phagemid carrying sgRNA4 (prom. J23110, backbone pBR322)	235469	This paper
pHK302.5	pBR322 ori, F1 ori, Amp^R, J23110 – sgRNA5	ampicillin	Message phagemid carrying sgRNA5 (prom. J23110, backbone pBR322)	235470	This paper
pHK302.6	pBR322 ori, F1 ori, Amp^R, J23110 – sgRNA6	ampicillin	Message phagemid carrying sgRNA6 (prom. J23110, backbone pBR322)	235471	This paper
pHK052.3	RSF1030 ori, F1 ori, Gm^R, J23119 – sgRNA3	gentamicin	Message phagemid carrying sgRNA3 (prom. J23119, backbone RSF1030)	235472	This paper
pHK352.3	RSF1030 ori, F1 ori, Gm^R, J23110 – sgRNA3	gentamicin	Message phagemid carrying sgRNA3 (prom. J23110, backbone RSF1030)	235473	This paper
pHK316	pCDF ori, Spec^R, *tetR*, Ptet – (M13) gene VIII	spectinomycin	Inducible gene VIII by Ptet promoter	235474	This paper
pHK326	pCDF ori, Spec^R, *lacI*, Ptac – (M13) gene VIII	spectinomycin	Inducible gene VIII by Ptac promoter	235475	This paper
pHK336	pCDF ori, Spec^R, *luxR*, Plux – (M13) gene VIII	spectinomycin	Inducible gene VIII by Plux promoter	235476	This paper
pHK346	pCDF ori, Spec^R, *araC*, Pbad – (M13) gene VIII	spectinomycin	Inducible gene VIII by Pbad promoter	235477	This paper
pHK318	pCDF ori, Spec^R, *luxR*, *tetR*, Plux – tetO – (M13) gene VIII	spectinomycin	Inducible gene VIII by Plux/tet hybrid promoter	235478	This paper
pHK328	pCDF ori, Spec^R, *araC*, *lacI*, Pbad – lacO – (M13) gene VIII	spectinomycin	Inducible gene VIII by Pbad/lac hybrid promoter	235479	This paper
pHK101	pCDF ori, Spec^R, *tetR*, **node 1:** Ptac – cI, **node 2:** J23119 – bs cI – (M13) gene VIII	spectinomycin	Repression of gene VIII by Ptet promoter	235480	This paper
pHK201	pCDF ori, Spec^R, *luxR*, **node 1:** Ptet – cI, **node 2:** J23119 – bs cI – (M13) gene VIII	spectinomycin	Repression of gene VIII by Ptac promoter	235481	This paper
pHK401	pCDF ori, Spec^R, *luxR*, *tetR*, **node 1:** Plux – tetO – cI, **node 2:** J23119 – bs cI – (M13) gene VIII	spectinomycin	Repression of gene VIII by Plux/tet hybrid promoter	235482	This paper
pHK501	pCDF ori, Spec^R, *araC*, *lacI*, **node 1:** Pbad – lacO – cI, **node 2:** J23119 – bs cI – (M13) gene VIII	spectinomycin	Repression of gene VIII by Pbad/lac hybrid promoter	235483	This paper
pHK001.23	pColA ori, Kan^R, **node 2:** J23100 – bs2- sfGFP , **node 3:** J23119 – bs3 - mCherry	kanamycin	dual NOT gate receiver with bs for sgRNA2 and sgRNA3	235484	This paper
pHK001.3QS	pColA ori, Kan^R, **node 1:** *cinR*, **node 2:** Pcin – sgRNA3, **node 3:** J23119 – bs3 - mCherry	kanamycin	inducible NOT gate receiver with bs for sgRNA3	235485	This paper
pHK-cinI	pCDF, Spec^R, *lacI*, Ptac - *cinI*	spectinomycin	Inducible gene cinI for OC14-HSL production by Ptac promoter	235486	This paper

Table S3. Oligonucleotides used in this study

oligo	sequence 5'->3'	note	circuit
PR_L_166	CAATAAACAGTTGATAGGGCTTCTCCGTTACAGCCTGCGGTCCGG	linker 0 - prefix	logic gates (receiver)
PR_L_167	TAACGGAGAAGCCCTATCAACTGTTTATTGCGGGCGTCCCAGCGA	linker 0 - suffix	logic gates (receiver)
PR_L_4	GGGACTACACTTACGAAACTATTGATTGCTCAGCCTGCGGTCCGG	linker 1 - prefix	logic gates (receiver)
PR_L_3	AGCAATCAATAGTTTCGTAAGTGTAGTCCCCGGGCGTCCCAGCGA	linker 1 - suffix	logic gates (receiver)
PR_L_168	AAGAGATTTCTACACGATTGAGCACTGTCTCAGCCTGCGGTCCGG	linker 10 - prefix	logic gates (receiver)
PR_L_169	AGACAGTGCTCAATCGTGTAGAAATCTCTTCGGGCGTCCCAGCGA	linker 10 - suffix	logic gates (receiver)
PR_L_170	CTATTACACTCGTCGTTGGAAACTGAAGATCAGCCTGCGGTCCGG	linker 11 - prefix	logic gates (receiver)
PR_L_161	ATCTTCAGTTTCCAACGACGAGTGTAATAGCGGGCGTCCCAGCGA	linker 11 - suffix	logic gates (receiver)
PR_L_43	ATAGGATAGATTCTGGAAACTTTACCGTCCCAGCCTGCGGTCCGG	linker 14 - prefix	logic gates (receiver)
PR_L_305	GGACGGTAAAGTTTCCAGAATCTATCCTATCGGGCGTCCCAGCGA	linker 14 - suffix	logic gates (receiver)
PR_L_6	GTAGTGCTTATCAGACCCAATACTGTTGAACAGCCTGCGGTCCGG	linker 17 - prefix	logic gates (receiver)
PR_L_5	TTCAACAGTATTGGGTCTGATAAGCACTACCGGGCGTCCCAGCGA	linker 17 - suffix	logic gates (receiver)
PR_L_8	TGAACAGTTGCTCTGATTGAAACCACGATTCAGCCTGCGGTCCGG	linker 19 - prefix	logic gates (receiver)
PR_L_7	AATCGTGGTTTCAATCAGAGCAACTGTTCACGGGCGTCCCAGCGA	linker 19 - suffix	logic gates (receiver)
PR_L_162	AGAGCCGAATCGCACTTATTTACAGTAGTTCAGCCTGCGGTCCGG	linker 21 - prefix	logic gates (receiver)
PR_L_163	AACTACTGTAAATAAGTGCGATTCGGCTCTCGGGCGTCCCAGCGA	linker 21 - suffix	logic gates (receiver)
PR_L_164	TAGAACTCTGTGAGGATAAAGTCTCCCTTACAGCCTGCGGTCCGG	linker 22 - prefix	logic gates (receiver)
PR_L_165	TAAGGGAGACTTTATCCTCACAGAGTTCTACGGGCGTCCCAGCGA	linker 22 - suffix	logic gates (receiver)
PR_L_18	AGATAGCCGTTACACAGGTGACACTTATTTCAGCCTGCGGTCCGG	linker 24 - prefix	logic gates (receiver)
PR_L_17	AAATAAGTGTCACCTGTGTAACGGCTATCTCGGGCGTCCCAGCGA	linker 24 - suffix	logic gates (receiver)
PR_L_22	AGTGACGACTGCGAAGTAACCTCTATTTATCAGCCTGCGGTCCGG	linker 25 - prefix	logic gates (receiver)
PR_L_21	ATAAATAGAGGTTACTTCGCAGTCGTCACTCGGGCGTCCCAGCGA	linker 25 - suffix	logic gates (receiver)
PR_C_15	ACGAGTCACTGTTGAGGATAAATACTTTCTCTACTA	pC F	logic gates (receiver)
PR_C_13	GGACGGTAAAGTTTCCAGAATCTATCCTAT	pC R	logic gates (receiver)
PR_TU_16	ATAGGATAGATTCTGGAAACTTTACCGTCC	TU - 1 F	logic gates (receiver)
PR_TU_3	ATTTTCTACAGTCTCAGTTTCAGGGATTTC	TU - 1 R	logic gates (receiver)
PR_TU_17	GAAATCCCTGAAACTGAGACTGTAGAAAAT	TU - 2 F	logic gates (receiver)
PR_TU_18	TGAGTTTTCAACGATTACCAATACTTCT	TU - 2 R	logic gates (receiver)
PR_TU_19	AGAAGTATTGGTAATCGTTGAAAACTCA	TU - 3 F	logic gates (receiver)
PR_TU_20	TAGTAGAGAAAGTATTTATCCTCAACAGTGACTCGT	TU - 3 R	logic gates (receiver)
PR_P_314	GCTAGCATTGTACCTAGGACTGAGCTAGCCGTAAAACTGGCCGTCGTTTTACA	J23110 - message phagemid backbone R	message phagemids
PR_P_164	GCTAGCATTATACCTAGGACTGAGCTAGCTGTCAAACTGGCCGTCGTTTTACA	J23119 - message phagemid backbone R	message phagemids
PR_P_207	GCTCGCTGGGACGCCCGATTCTGCAGATATCCATC	message phagemid backbone F	message phagemids
PR_P_313	TTTACGGCTAGCTCAGTCCTAGGTACAATGCTAGCCAGCCTGCGGTCCGG	sgRNA - prefix - J23110	message phagemids
PR_P_161	TTGACAGCTAGCTCAGTCCTAGGTATAATGCTAGCCAGCCTGCGGTCCGG	sgRNA - prefix - J23119	message phagemids
PR_P_204	GATGGATATCTGCAGAATCGGGCGTCCCAGCGAGC	sgRNA - suffix	message phagemids
PR_P_482	CTCGGTACCAAATTCCAGAAAAGAGG	pCDF F (single input)	inducible gVIII (single input)
PR_P_197	TTTTCTACAGTCTCAGTTTCAGGGATTTCG	pCDF R	inducible gVIII (single & dual input)
PR_P_610	TAATTTTGTTTAACTTTAAGAAGGAGATATACCATGAAAAAGTCTTTAGCCCTCAAAGC	M13 gene VIII F + RBS	inducible gVIII (single & dual input)
PR_P_609	CCTCTTTTCTGGAATTTGGTACCGAGTCAGCTTGCTTTCGAGGTGAATTTC	M13 gene VII R	inducible gVIII (single & dual input)
PR_P_388	GAAATCCCTGAAACTGAGACTGTAGAAAATTTAGGACCCACTTTCACATTTAAG	TetR & Ptet F	inducible gVIII (single input)
PR_P_479	GAAATCCCTGAAACTGAGACTGTAGAAAATTCACTGCCCGCTTTCCAGTCG	LacI & Ptac F	inducible gVIII (single input)
PR_P_484	GAAATCCCTGAAACTGAGACTGTAGAAAATTTATTAATTTTTGAAGTACGGACAGTCG	LuxR & Plux F	inducible gVIII (single & dual input)
PR_P_486	GAAATCCCTGAAACTGAGACTGTAGAAAATTTATGACAACTTGACGGCTACATCATTC	AraC & Pbad F	inducible gVIII (single & dual input)
PR_P_582	TCATGGTATATCTCCTTCTTAAAGTTAAACAAAATTA	promoter R	inducible gVIII (single & dual input) & inverter gVIII (single & dual input)
PR_P_50	AGGTGGCACTTTTCGGGGAAATGTGCGAGTCACTGCATCCTAGGAG	pCDF F (dual input)	inducible gVIII (dual input)

PR_P_625	CACATTTCCCCGAAAAGTGCCACCTTTAGGACCCACTTTCACATTTAAGTTG	TetR F	inducible gVIII (dual input)
PR_P_624	GTAAAAGACGCAGTGACGGCGCAGTGAGCGTACTGTCAC	TetR R	inducible gVIII (dual input)
PR_P_622	GCCGTCACTGCGTCTTTTACTCACACTGGCTCACCTTCGGGTGGG	double terminator B0014 F	inducible gVIII (dual input)
PR_P_621	ATTTTCTACAGTCTCAGTTTCAGGGATTTCGAATTCAAATAATAAAAAAGCCGGATTAATAATCTGGC	double terminator B0014 R	inducible gVIII (dual input)
PR_P_442	TAACGGAGAAGCCCTATCAACTGTTTATTGACCTGTACGATCCTACAGGTGCTTATG	LuxR R	inducible gVIII (dual input)
PR_P_626	CAGCCTGCGGTCCGGACCTGTAGGATCGTACAGGTTTACGCAAGAAAATGGTTTGTTATAGTCGAATATCCCTA TCAGTGATAGAGATCGCTGGGACGCCCG	Plux + tetO with prefix & suffix	inducible gVIII (dual input)
PR_P_623	CACATTTCCCCGAAAAGTGCCACCTTCACTGCCCGCTTTCCAGTC	LacI F	inducible gVIII (dual input)
PR_P_400	GTAAAAGACGCAGTGACGGC	LacI R	inducible gVIII (dual input)
PR_P_402	TATGGAGAAACAGTAGAGAGTTGCG	AraC R	inducible gVIII (dual input)
PR_P_619	CTGACGCTTTTTATCGCAACTCTCTACTGTTTCTCCATACCGGAATTGTGAGCGGATAACAA TTTAATTTTGTTTAACTTTAAGAAGGAGATATACC	Pbad + lacO + RBS	inducible gVIII (dual input)
PR_P_630	AGACAGTGCTCAATCGTGTAGAAATCTCTTAATTGTTATCCGCTCACAATTCC	lacO R	inverter gVIII (single & dual input)
PR_P_629	AGACAGTGCTCAATCGTGTAGAAATCTCTTGTGCTCATTATCTCTATCACTGATAGGG	Ptet R	inverter gVIII (single & dual input)
PR_P_711	CAGCCTGCGGTCCGGCTGCCGTATAGGCAGAAAGAGGAGAAAATGAGCACAAAAAAGAAACCATTAA CACAAGAGCAG	cI F with prefix and RBS	inverter gVIII (single & dual input)
PR_P_712	CGGGCGTCCCAGCGACTGCCTATACGGCAGTGAACTCAAGCTGCTAAAGCGTAGTTTTCGTCGTTTGC	cI R with suffix	inverter gVIII (single & dual input)

Table S4. Synthetic biology parts used to construct plasmids (5' -> 3')

Part name	Sequence
sgRNA1	CAGCCTGCGGTCCGGGTTCACTGCCGTATAGGCAGTCTCAAGCTAGACTCTAGTGGTTTCAGAGCTATGCTGGAAACAGCATAGCAAGTTGAAATAAG<br>GCTAGTCCGTTATCAACTTGAAAAAGTGGCACCGAGTCGGTGCGTTCACTGCCGTATAGGCAGTCGCTGGGACGCCCG
sgRNA2	CAGCCTGCGGTCCGGGTTCACTGCCGTATAGGCAGATCAGTGTGTACTAAGTACTGTTTCAGAGCTATGCTGGAAACAGCATAGCAAGTTGAAATAAG<br>GCTAGTCCGTTATCAACTTGAAAAAGTGGCACCGAGTCGGTGCGTTCACTGCCGTATAGGCAGTCGCTGGGACGCCCG
sgRNA3	CAGCCTGCGGTCCGGGTTCACTGCCGTATAGGCAGTGACTGAGCTAGTGTACTCTGTTTCAGAGCTATGCTGGAAACAGCATAGCAAGTTGAAATAAG<br>GCTAGTCCGTTATCAACTTGAAAAAGTGGCACCGAGTCGGTGCGTTCACTGCCGTATAGGCAGTCGCTGGGACGCCCG
sgRNA4	CAGCCTGCGGTCCGGGTTCACTGCCGTATAGGCAGGACACATCTTAGAGTATGTAGTTTCAGAGCTATGCTGGAAACAGCATAGCAAGTTGAAATAAG<br>GCTAGTCCGTTATCAACTTGAAAAAGTGGCACCGAGTCGGTGCGTTCACTGCCGTATAGGCAGTCGCTGGGACGCCCG
sgRNA5	CAGCCTGCGGTCCGGGTTCACTGCCGTATAGGCAGAAGTGAGTCTGAGCTTAGATGTTTCAGAGCTATGCTGGAAACAGCATAGCAAGTTGAAATAAG<br>GCTAGTCCGTTATCAACTTGAAAAAGTGGCACCGAGTCGGTGCGTTCACTGCCGTATAGGCAGTCGCTGGGACGCCCG
sgRNA6	CAGCCTGCGGTCCGGGTTCACTGCCGTATAGGCAGTTTAGTAGTCTACTTAGATGGTTTCAGAGCTATGCTGGAAACAGCATAGCAAGTTGAAATAAG<br>GCTAGTCCGTTATCAACTTGAAAAAGTGGCACCGAGTCGGTGCGTTCACTGCCGTATAGGCAGTCGCTGGGACGCCCG
bs1 (antisense)	CAGCCTGCGGTCCGGCCACACTAGAGTCTAGCTTGAGATCGCTGGGACGCCCG
bs2 (antisense)	CAGCCTGCGGTCCGGCCAAGTACTTAGTACACACTGATTCGCTGGGACGCCCG
bs3 (antisense)	CAGCCTGCGGTCCGGCCAAGAGTACACTAGCTCAGTCATCGCTGGGACGCCCG
bs4 (antisense)	CAGCCTGCGGTCCGGCCATACATACTCTAAGATGTGTCTCGCTGGGACGCCCG
bs5 (antisense)	CAGCCTGCGGTCCGGCCAATCTAAGCTCAGACTCACTTTCGCTGGGACGCCCG
bs6 (antisense)	CAGCCTGCGGTCCGGCCACATCTAAGTAGACTACTAAATCGCTGGGACGCCCG
Prom. J23100	CAGCCTGCGGTCCGGTTGACGGCTAGCTCAGTCCTAGGTACAGTGCTAGCTCGCTGGGACGCCCG
Prom. J23108	CAGCCTGCGGTCCGGCTGACGGCTAGCTCAGTCCTAGGTATAATGCTAGCTCGCTGGGACGCCCG
Prom. J23110	CAGCCTGCGGTCCGGTTTACGGCTAGCTCAGTCCTAGGTACAATGCTAGCTCGCTGGGACGCCCG
Prom. J23115	CAGCCTGCGGTCCGGTTTATGGCTAGCTCAGCCCTTGGTACAATGCTAGCTCGCTGGGACGCCCG
Prom. J23119	CAGCCTGCGGTCCGGTTGACAGCTAGCTCAGTCCTAGGTATAATGCTAGC TCGCTGGGACGCCCG
Ptet	CAGCCTGCGGTCCGGTTTTTTCCCTATCAGTGATAGAGATTGACATCCCTATCAGTGATAGAGATAATGAGCACTCGCTGGGACGCCCG
Ptac	CAGCCTGCGGTCCGGGTTGACAATTAATCATCGGCTCGTATAATGTGTGGAATTGTGAGCGCTCACAATT TCGCTGGGACGCCCG
Plux	CAGCCTGCGGTCCGGACCTGTAGGATCGTACAGGTTTACGCAAGAAAATGGTTTGTTATAGTCGAATAAA TCGCTGGGACGCCCG
Pbad	ACTTTTCATACTCCCGCCATTCAGAGAAGAAACCAATTGTCCATATTGCATCAGACATTGCCGTCACTGCGTCTTTTACTGGCTCTTCTCGCTAACCAAACCG<br>GTAACCCCGCTTATTAAAAGCATTCTGTAACAAAGCGGGACCAAAGCCATGACAAAAACGCGTAACAAAAGTGTCTATAATCACGGCAGAAAAGTCCACAT<br>TGATTATTTGCACGGCGTCACACTTTGCTATGCCATAGCATTTTTATCCATAAGATTAGCGGTTCCTACCTGACGCTTTTTATCGCAACTCTCTACTGTTTCTCCA<br>TACC
Plux/tet	CAGCCTGCGGTCCGGACCTGTAGGATCGTACAGGTTTACGCAAGAAAATGGTTTGTTATAGTCGAATATCCCTATCAGTGATAGAGATCGCTGGGACGCCCG
Pbad/lac	ACTTTTCATACTCCCGCCATTCAGAGAAGAAACCAATTGTCCATATTGCATCAGACATTGCCGTCACTGCGTCTTTTACTGGCTCTTCTCGCTAACCAAA<br>CCGGTAACCCCGCTTATTAAAAGCATTCTGTAACAAAGCGGGACCAAAGCCATGACAAAAACGCGTAACAAAAGTGTCTATAATCACGGCAGAAAAG<br>TCCACATTGATTATTTGCACGGCGTCACACTTTGCTATGCCATAGCATTTTTATCCATAAGATTAGCGGTTCCTACCTGACGCTTTTTATCGCAACTCTCTA<br>CTGTTTCTCCATACCGGAATTGTGAGCGGATAACAATT
tetR (reverse orientation) + Ptet	GAAATCCCTGAAACTGAGACTGTAGAAAATTTAGGACCCACTTTCACATTTAAGTTGTTTTTCTAATCCGCATATAATCAATTCAAGGCCGAATAAGAAG<br>GCTGGCTCTGCACCTTGGTGATCAAATAATTCGATAGCTTGTCGTAATAATGGCGGCATACTATCAGTAGTAGGTGTTTCCCTTTCTTCTTTAGCGACTTG<br>ATGCTCTTGATCTTCCAATACGCAACCTAAAGTAAAATGCCCCACAGCGCTGAGTGCATATAATGCATTCTCTAGTGAAAAACCTTGTTGGCATAAAAAG<br>GCTAATTGATTTTCGAGAGTTTCATACTGTTTTTCTGTAGGCCGTGTACCTAAATGTACTTTTGCTCCATCGCGATGACTTAGTAAAGCACATCTAAAACT<br>TTTAGCGTTATTACGTAAAAAATCTTGCCAGCTTTCCCCTTCTAAAGGGCAAAAGTGAGTATGGTGCCTATCTAACATCTCAATGGCTAAGGCGTCGAGC<br>AAAGCCCGCTTATTTTTTACATGCCAATACAATGTAGGCTGCTCTACACCTAGCTTCTGGGCGAGTTTACGGGTTGTTAAACCTTCGATTCCGACCTCATT<br>AAGCAGCTCTAATGCGCTGTTAATCACTTTACTTTTATCTAATCTGGACATTTTTTTCCTCCTGGATCCTTAATTTTTAAAGTATGGGCAATCAATTGCTCCT<br>GTTAAAATTGCTTTAGAAATACTTTGGCAGCGGTTTGTTGTATTGAGTTTCATTTGCGCATTGGTTAAATGGAAAGTGACAGTACGCTCACTGCGAATTC<br>GAGTTGTAAAACGACGGCCAGTACTCCACCGTTGGCTTTTTTCCCTATCAGTGATAGAGATTGACATCCCTATCAGTGATAGAGATAATGAGCACTAATT<br>TTGTTTAACTTTAAGAAGGAGATATACC
lacI (reverse orientation) + Ptac	GAAATCCCTGAAACTGAGACTGTAGAAAATTCACTGCCCGCTTTCCAGTCGGGAAACCTGTCGTGCCAGCTGCATTAATGAATCGGCCAACGCGCGGG<br>GAGAGGCGGTTTGCGTATTGGGCGCCAGGGTGGTTTTTCTTTTCACCAGTGAGACTGGCAACAGCTGATTGCCCTTCACCGCCTGGCCCTGAGAGAG<br>TTGCAGCAAGCGGTCCACGCTGGTTTGCCCCAGCAGGCGAAAATCCTGTTTGATGGTGGTTAACGGCGGGATATAACATGAGCTATCTTCGGTATCGTC<br>GTATCCCACTACCGAGATATCCGCACCAACGCGCAGCCCGGACTCGGTAATGGCGCGCATTGCGCCCAGCGCCATCTGATCGTTGGCAACCAGCATCGC<br>AGTGGGAACGATGCCCTCATTCAGCATTTGCATGGTTTGTTGAAAACCGGACATGGCACTCCAGTCGCCTTCCCGTTCCGCTATCGGCTGAATTTGATTG<br>CGAGTGAGATATTTATGCCAGCCAGCCAGACGCAGACGCGCCGAGACAGAACTTAATGGGCCCGCTAACAGCGCGATTTGCTGGTGACCCAATGCGAC<br>CAGATGCTCCACGCCCAGTCGCGTACCGTCCTCATGGGAGAAAATAATACTGTTGATGGGTGTCTGGTCAGAGACATCAAGAAATAACGCCGGAACATTA<br>GTGCAGGCAGCTTCCACAGCAATGGCATCCTGGTCATCCAGCGGATAGTTAATGATCAGCCCACTGACGCGTTGCGCGAGAAGATTGTGCACCGCCGCT<br>TTACAGGCTTCGACGCCGCTTCGTTCTACCATCGACACCACCACGCTGGCACCCAGTTGATCGGCGCGAGATTTAATCGCCGCGACAATTTGCGACGGCG<br>CGTGCAGGGCCAGACTGGAGGTGGCAACGCCAATCAGCAACGACTGTTTGCCCGCCAGTTGTTGTGCCACGCGGTTGGGAATGTAATTCAGCTCCGCC<br>ATCGCCGCTTCCACTTTTTCCCGCGTTTTCGCAGAAACGTGGCTGGCCTGGTTCACCACGCGGGAAACGGTCTGATAAGAGACACCGGCATACTCTGCG<br>ACATCGTATAACGTTACTGGTTTCATATTCACCACCCTGAATTGACTCTCTTCCGGGCGCTATCATGCCATACCGCGAAAGGTTTTGCGCCATTCGATGGCG<br>CGCCGCCATTGCGGCCGCGCCGTCACTGCGTCTTTTACGAGCTGTTGACAATTAATCATCGGCTCGTATAATGTGTGGAATTGTGAGCGCTCACAATTTAA<br>TTTTGTTTAACTTTAAGAAGGAGATATACC
luxR (reverse orientation) + Plux	GAAATCCCTGAAACTGAGACTGTAGAAAATTTATTAATTTTTGAAGTACGGACAGTCGATAGCGCCGGTCAGGATTGCTTTGGAGATGGATTGGCAGCG<br>ATTCGTGGTGTTCAGTTTCATTTGCGCGTTCGTCAGATGAAACGTAACGGTACGTTCGGAACAACCCAGGATTTTACTAATATCCCAAGAGCTTTTGCCTT<br>CACAGGCCCACGCCAGGCATTCTTTTTCGCGTTTGGTCAGGTCATTGTTACTTTTGTTGTTAGCGATGTTGATTTTACGGTAGTTATCAACCAGAGACGGC<br>ACGATCAGCGGAATATTCATGCATGCATGCAGGAACAGGCTGTCAATATAGTTATCTTTTTCAGAGTGAGCGAAGCTCAGCATACCAAAGCCATTGTTTGC<br>GGTATGGATCGGGAACGAAAAACCCGTAATCAGGCCGCTGGTTTTGGCTTCTTTGATCACGTTCGGAGATTTTTTGTTGACCGCATTGTTTTCAAAGATAT<br>TCCAGTTAATCGGGGAGTGATTGGAGTTACTGTAGTCCACGATCGGATCATATTTAATCAGATTGGCGTCATCGTAATACTGACGCCATTTTTTCGGGTAGT<br>TGTCCAGGATCGAAATATCTGATTTGACCATACTGTGCGGATAGATAATCGCCAGCAGGTAATATTCGCAATGAACCATTTTGGTCATATCGCTCAGGCACTG<br>GTTGATGTCGTTGTTTGAGCGACAAGCTTTGATTTTGTTGATGATGCGGTACGTATCGTCGGCGTTGATGTTTTTCATACCCATCTCTTTATCCTTACCTATTG<br>TTTGTCGCAAGTTTTGCGTGTTATATATCATTAAAACGGTAATGGATTGACATTTGATTCTAATAAATTGGATTTTTGTCACACTATTGTATCGCTGGGAATAC<br>AATTACTTAACATAAGCACCTGTAGGATCGTACAGGTCAATAAACAGTTGATAGGGCTTCTCCGTTAACCTGTAGGATCGTACAGGTTTACGCAAGAAAATG<br>GTTTGTTATAGTCGAATAAATAATTTTGTTTAACTTTAAGAAGGAGATATACC

araC (reverse orientation) + Pbad	GAAATCCCTGAAACTGAGACTGTAGAAAATTTATGACAACTTGACGGCTACATCATTCACTTTTTCTTCACAACCGGCACGGAACTCGCTCGGGCTGGCCC<br>CGGTGCATTTTTTAAATACCCGCGAGAAATAGAGTTGATCGTCAAAACCAACATTGCGACCGACGGTGGCGATAGGCATCCGGGTGGTGCTCAAAAGCAG<br>CTTCGCCTGGCTGATACGTTGGTCCTCGCGCCAGCTTAAGACGCTAATCCCTAACTGCTGGCGGAAAAGATGTGACAGACGCGACGGCGACAAGCAAACA<br>TGCTGTGCGACGCTGGCGATATCAAAATTGCTGTCTGCCAGGTGATCGCTGATGTACTGACAAGCCTCGCGTACCCGATTATCCATCGGTGGATGGAGCGA<br>CTCGTTAATCGCTTCCATGCGCCGCAGTAACAATTGCTCAAGCAGATTTATCGCCAGCAGCTCCGAATAGCGCCCTTCCCCTTGCCCGGCGTTAATGATTTGC<br>CCAAACAGGTCGCTGAAATGCGGCTGGTGCGCTTCATCCGGGCGAAAGAACCCCGTATTGGCAAATATTGACGGCCAGTTAAGCCATTCATGCCAGTAGG<br>CGCGCGGACGAAAGTAAACCCACTGGTGATACCATTCGCGAGCCTCCGGATGACGACCGTAGTGATGAATCTCTCCTGGCGGGAACAGCAAAATATCACC<br>CGGTCGGCAAACAAATTCTCGTCCCTGATTTTTCACCACCCCCTGACCGCGAATGGTGAGATTGAGAATATAACCTTTCATTCCCAGCGGTCGGTCGATAAA<br>AAAATCGAGATAACCGTTGGCCTCAATCGGCGTTAAACCCGCCACCAGATGGGCATTAAACGAGTATCCCGGCAGCAGGGGATCATTTTGCGCTTCAGCCA<br>TACTTTTCATACTCCCGCCATTCAGAGAAGAAACCAATTGTCCATATTGCATCAGACATTGCCGTCACTGCGTCTTTTACTGGCTCTTCTCGCTAACCAAACCG<br>GTAACCCCGCTTATTAAAAGCATTCTGTAACAAAGCGGGACCAAAGCCATGACAAAAACGCGTAACAAAAGTGTCTATAATCACGGCAGAAAAGTCCACAT<br>TGATTATTTGCACGGCGTCACACTTTGCTATGCCATAGCATTTTTATCCATAAGATTAGCGGTTCCTACCTGACGCTTTTTATCGCAACTCTCTACTGTTTCTCCA<br>TACCTAATTTTGTTTAACTTTAAGAAGGAGATATACC
cinR (reverse orientation) + Pcin	TTACCAATTACGTCGCGTCATGCGGATCCTATAGGGATTGATGATGCGCAATTGAACAGCCCGCGACGCGGCGGCCGAGATCGTGGTGCAGCCGAGCCTGAA<br>GCGGGCGGTTTTCAGGTAATCGCGTGTGGTATGCTCTGATATGCCCAGGATGACCGAAATATCCTTGTAATCCTTGCCGAGGGCGGTCCAGTGCAGACACTC<br>GATCTCGCGCGGCGACAATGCCGGCACCGGATCGTTTTCGCCATGCAGCTCATATACGGCCTTGCGGTGGATCAGATGGGCGATCTCGATCCACTCATTGCG<br>GCAGCGGCGCACGAGCTCGGTCCATTCGTCGGCCGGTATATGGGCATTCAGCGACAGCAGGGCGCGGCGCTGCGCCTTGTCGGCGACGGGGATGGAGTAG<br>CCATTGTCATCGATGCCGTGTTTCTGGGCGTCGACCAGCATGGCATAGGCCTCCGGCGTCGGTTCGACCTCGCTCCAGTCGAAGGGCAGCTGGCGTTCGAA<br>GCCCTGCTTGATGATCGGATCGACCTTCACATAGCAGTTGAGGAGGTAACGGGAAACCCAGGCATCCGGATAGGTGGTGCGCACGAAGGGCGAATCGATCT<br>TGCTCGCGATTGTCTGGGCGAGATGGTAGGTGACGAAATCGAGGTTATATTCCGCCTGGAGAATACGGATGGCGGCATCCACGTTGGCCGCCGCTTTGATCT<br>GTTCGAACGCGGACTCGAACTTTTCGCTATAGGTATTCTCAATCATATTCACCACCCTGAATTGACTCTCTTCCGGGCGCTATCATGCCATACCGCGAAAGGTTT<br>TGCGCCATTCGATGGCGCGCCGCCATTGGACCAAAACGAGAGCCGAATCGCACTTATTTACAGTAGTTCAGCCTGCGGTCCGGGACATGCTCTGATCCCCCTC<br>ATCTGGGGGGGCCTATCTGAGGGAATTTCCGATCCGGCTCGCCTGAACCATTCTGCTTTCCACGAACTTGAAAACGCTAGTCGCTGGGACGCCCG
cinI	CAGCCTGCGGTCCGGCTGCCGTATAGGCAGAAAGAGGAGAAAATGCGGGGGAGTGTTACTAGTATGTTCGTTATCATTCAGGCACATGAGTATCAGAAATAC<br>GCTGCCGTACTCGACCAGATGTTTCGTCTGCGCAAGAAGGTCTTCGCCGATACGCTCTGCTGGGACGTTCCTGTCATCGGCCCTTACGAACGTGACAGCTAC<br>GATTCGCTTGCTCCCGCCTATCTCGTCTGGTGCAACGACAGCCGCACCCGTCTTTATGGCGGCATGCGCCTGATGCCGACGACCGGCCCGACCCTTCTCTACG<br>ACGTCTTCCGCGAGACGTTCCCTGATGCCGCCGATCTTATCGCCCCCGGCATCTGGGAAGGCACGCGCATGTGCATCGACGAGGAGGCGATCGCCAAGGAT<br>TTCCCCGAGATCGACGCCGGCCGCGCCTTCTCCATGATGCTGCTCGCGCTTTGCGAATGCGCGCTCGATCACGGCATCCACACGATGATCTCCAACTACGAGC<br>CCTACCTCAAGCGCGTCTACAAGCGCGCCGGCGCCGAGGTGGAAGAACTCGGCCGCGCAGACGGCTACGGCAAATATCCCGTCTGCTGCGGCGCCTTCGA<br>AGTCTCGGACCGCGTGCTGCGCAAGATGCGCGCCGCCCTCGGCCTCACCCTACCCCTTTATGTCAGGCACGTGCCGGCCCGCTCGGTCGTGACCCAATTCCT<br>GGAGATGGCAGCAGCAGCGAACGACGAAAATTACGCCCTTGCAGCGTAATAAAGTTCACTGCCGTATAGGCAGTCGCTGGGACGCCCG
M13 gene VIII	CAGCCTGCGGTCCGGGTTCACTGCCGTATAGGCAGTAATTTTGTTTAACTTTAAGAAGGAGATATACCATGAAAAAGTCTTTAGCCCTCAAAGCCTCTGTAG<br>CCGTTGCTACCCTCGTTCCGATGCTGTCTTTCGCTGCTGAGGGTGACGATCCCGCAAAAGCGGCCTTTAACTCCCTGCAAGCCTCAGCGACCGAATATATCG<br>GTTATGCGTGGGCGATGGTTGTTGTCATTGTCGGCGCAACTATCGGTATCAAGCTGTTTAAGAAATTCACCTCGAAAGCAAGCTGAGTTCACTGCCGTATAG<br>GCAGTCGCTGGGACGCCCG
cI	CAGCCTGCGGTCCGGGTTCACTGCCGTATAGGCAGAAAGAGGAGAAAATGAGCACAAAAAAGAAACCATTAACACAAGAGCAGCTTGAGGACGCACGTC<br>GCCTTAAAGCAATTTATGAAAAAAAGAAAAATGAACTTGGCTTATCCCAGGAATCTGTCGCAGACAAGATGGGGATGGGGCAGTCAGGCGTTGGTGCTTTA<br>TTTAATGGCATCAATGCATTAAATGCTTATAACGCCGCATTGCTTGCAAAAATTCTCAAAGTTAGCGTTGAAGAATTTAGCCCTTCAATCGCCAGAGAAATCTAC<br>GAGATGTATGAAGCGGTTAGTATGCAGCCGTCACTTAGAAGTGAGTATGAGTACCCTGTTTTTTCTCATGTTCAGGCAGGGATGTTCTCACCTGAGCTTAGAA<br>CCTTTACCAAAGGTGATGCGGAGAGATGGGTAAGCACAACCAAAAAAGCCAGTGATTCTGCATTCTGGCTTGAGGTTGAAGGTAATTCCATGACCGCACCA<br>ACAGGCTCCAAGCCAAGCTTTCCTGACGGAATGTTAATTCTCGTTGACCCTGAGCAGGCTGTTGAGCCAGGTGATTTCTGCATAGCCAGACTTGGGGGTGAT<br>GAGTTTACCTTCAAGAAACTGATCAGGGATAGCGGTCAGGTGTTTTTACAACCACTAAACCCACAGTACCCAATGATCCCATGCAATGAGAGTTGTTCCGTTG<br>TGGGGAAAGTTATCGCTAGTCAGTGGCCTGAAGAGACGTTTGGCGCAGCAAACGACGAAAACTACGCTTTAGCAGCTTGAGTTCACTGCCGTATAGGCAGT<br>CGCTGGGACGCCCG
bs cI	CAGCCTGCGGTCCGGTAACACCGTGCGTGTTGACTATTTTACCTCTGGCGGTGATAATGGTTGCTCGCTGGGACGCCCG
sfGFP	CAGCCTGCGGTCCGGGTTCACTGCCGTATAGGCAGTAATTTTGTTTAACTTTAAGAAGGAGATATACATATGCGTAAAGGCGAAGAACTGTTTACCGGTGTGG<br>TTCCGATTCTGGTGGAACTGGACGGCGATGTTAATGGTCATAAATTCAGTGTTCGCGGCGAAGGTGAAGGCGATGCGACGAACGGCAAACTGACCCTGAAA<br>TTTATCTGCACCACGGGTAAACTGCCGGTCCCGTGGCCGACGCTGGTGACCACGCTGACCTATGGCGTTCAATGTTTTGCGCGTTACCCGGATCACATGAAAC<br>AGCACGACTTTTTCAAATCGGCCATGCCGGAAGGCTATGTGCAGGAACGTACGATTAGCTTTAAAGACGATGGTACGTATAAAACCCGCGCGGAAGTGAAAT<br>TCGAAGGCGATACCCTGGTTAACCGTATCGAACTGAAAGGTATCGATTTCAAAGAAGACGGCAATATTCTGGGTCATAAACTGGAATATAACTTCAATTCCCAC<br>AACGTGTACATCACCGCGGATAAACAGAAAAACGGCATTAAAGCCAATTTCAAAATCCGCCATAATGTGGAAGATGGTAGCGTTCAGCTGGCCGACCACTAT<br>CAGCAAAACACGCCGATTGGTGATGGCCCGGTCCTGCTGCCGGACAATCACTACCTGAGTACCCAGTCCGTGCTGTCAAAAGATCCGAACGAAAAACGTGA<br>CCACATGGTCCTGCTGGAATTTGTGACGGCTGCGGGTATCACCCACGGCATGGACGAACTGTATAAATAATAATCGCTGGGACGCCCG
mCherry	CAGCCTGCGGTCCGGTAATTTTGTTTAACTTTAAGAAGGAGATATACATATGGTGAGCAAGGGCGAGGAGGATAACATGGCCATCATCAAGGAGTTCATGCG<br>CTTCAAGGTGCACATGGAGGGCTCCGTGAACGGCCACGAGTTCGAGATCGAGGGCGAGGGCGAGGGCCGCCCCTACGAGGGCACCCAGACCGCCAAGCT<br>GAAGGTGACCAAGGGTGGCCCCCTGCCCTTCGCCTGGGACATCCTGTCCCCTCAGTTCATGTACGGCTCCAAGGCCTACGTGAAGCACCCCGCCGACATCCC<br>CGACTACTTGAAGCTGTCCTTCCCCGAGGGCTTCAAGTGGGAGCGCGTGATGAACTTCGAGGACGGCGGCGTGGTGACCGTGACCCAGGACTCCTCtCTGC<br>AGGACGGCGAGTTCATCTACAAGGTGAAGCTGCGCGGCACCAACTTCCCCTCCGACGGCCCCGTAATGCAGAAGAAGACtATGGGCTGGGAGGCCTCCTCC<br>GAGCGGATGTACCCCGAGGACGGCGCgCTGAAGGGCGAGATCAAGCAGAGGCTGAAGCTGAAGGACGGCGGCCACTACGACGCTGAaGTCAAGACCACC<br>TACAAGGCCAAGAAGCCCGTGCAGCTGCCCGGCGCgTACAACGTCAACATCAAGTTGGACATCACCTCCCACAACGAGGACTACACCATCGTGGAACAGTA<br>CGAACGCGCCGAGGGCCGCCACTCCACCGGCGGCATGGACGAGCTGTACAAGTAATCGCTGGGACGCCCG
pBR322 (ColE1) ori	TCATGACCAAAATCCCTTAACGTGAGTTTTCGTTCCACTGAGCGTCAGACCCCGTAGAAAAGATCAAAGGATCTTCTTGAGATCCTTTTTTTCTGCGCGTAATC<br>TGCTGCTTGCAAACAAAAAAACCACCGCTACCAGCGGTGGTTTGTTTGCCGGATCAAGAGCTACCAACTCTTTTTCCGAAGGTAACTGGCTTCAGCAGAGC<br>GCAGATACCAAATACTGTCCTTCTAGTGTAGCCGTAGTTAGGCCACCACTTCAAGAACTCTGTAGCACCGCCTACATACCTCGCTCTGCTAATCCTGTTACCAGT<br>GGCTGCTGCCAGTGGCGATAAGTCGTGTCTTACCGGGTTGGACTCAAGACGATAGTTACCGGATAAGGCGCAGCGGTCGGGCTGAACGGGGGGTTCGTGC<br>ACACAGCCCAGCTTGGAGCGAACGACCTACACCGAACTGAGATACCTACAGCGTGAGCTATGAGAAAGCGCCACGCTTCCCGAAGGGAGAAAGGCGGACA<br>GGTATCCGGTAAGCGGCAGGGTCGGAACAGGAGAGCGCACGAGGGAGCTTCCAGGGGGAAACGCCTGGTATCTTTATAGTCCTGTCGGGTTTCGCCACCT<br>CTGACTTGAGCGTCGATTTTTGTGATGCTCGTCAGGGGGGCGGAGCCTATGGAAAAACGCCAGCAACGCGGCCTTTTTACGGTTCCTGGCCTTTTGCTGGC<br>CTTTTGCTCACATGTTCTTTCCTGCGTTATCCCCTGATTCTGTGGATAACCGTATTACCGCCTTTGAGTGAGCTGATACCGCTCGCCGCAGCCGAACGACCGAG<br>CGCAGCGAGTCAGTGAGCGAGGAAGCGGAAGAGCGCCTGATGCGGTATTTTCTCCTTACGCATCTGTGCGGTATTTCACACCGCATATATGGTGCACTCTCA<br>GTACAATCTGCTCTGATGCCGCATAGTTAAGCCAGTATACACTCCGCTATCGCTACGTGACTGGGTCATGGCTGCGCCCCGACACCCGCCAACACCCGCTGAC<br>GCGCCCTGACGGGCTTGTCTGCTCCCGGCATCCGCTTACAGACAAGCTGTGACCGTCTCCGGGAGCTGCATGTGTCAGAGGTTTTCACCGTCATCACCGAAA<br>CGCGCGAGGCAGCTGCGGTAAAGCTCATCAGCGTGGTCGTGAAGCGATTCACAGATGTCTGCCTGTTCATCCGCGTCCAGCTCGTTGAGTTTCTCCAGAAGC<br>GTTAATGTCTGGCTTCTGATAAAGCGGGCCATGTTAAGGGCGGTTTTTTCCTGTTTGGTCACTGATGCCTCCGTGTAAGGGGGATTTCTGTTCATGGGGGTAA<br>TGATACCGATGAAACGAGAGAGGATGCTCACGATACGGGTTACTGATGATGAACATGCCCGGTTACTGGAACGTTGTGAGGGTAAACAACTGGCGGTATGG

	ATGCGGCGGGACCAGAGAAAAATCACTCAGGGTCAATGCCAGCGCTTCGTTAATACAGATGTAGGTGTTCCACAGGGTAGCCAGCAGCATCCTGCGATGCA<br>GATCCGGAACATAATGGTGCAGGG
RSF1030 ori	AACGGAATAGCTGTTCGTTGACTTGATAGACCGATTGATTCATCATCTCATAAATAAAGAAAAACCACCGCTACCAACGGTGGTTTTCTCAAGGTTCGCTGAG<br>CTACCAACTCTTTGAACCAAGGTAAGTGGGTTGGAGGACCGCACTCACCAAAATCTGTTCTTTCAGTTTAGCCTTAACAGGTGCATAACTTCAAGACAAAGTC<br>CTCTAAATCAGTTACCAATGGCTGCTGCCAGTGGCGATAAGTCGTGTCTTACCGGGTTGGACTCAAGACGATAGTTACCGGATAAGGCGCAGCGGTCGGGCT<br>GAACGGGGGGTTCGTGCACACAGCCCAGCTTGGAGCGAACGACCTACACCGAACTGAGATACCAACAGCGTGAGCTATGAGAAAGCGCCACGCTTCCCGA<br>AGGGAGAAAGGCGGACAGGTATCCGGTAAGCGGCAGGGTCGGAACAGGAGAGCGCACGAGGGAGCTTCCAGGGGGAAACGCCTGGTATCTTTATAGTCC<br>TGTCGGGTTTCGCCACCTCTGGCTTGAGCGTCGATTTTTGTGATGCTCGTCAGGGGGGCGGAGCCTATGGAAAAACGCCTGCGGCGTTGGCTTCTTCCGGT<br>GCTTTGCTTTTTGCTCACATGTTCTTTCCGGCTTTATCCCCTGATTCTGTGGATAACCGTATTACCGCTTTTGAGTGAGCTGACACCGCTCGCCGCAGTCGAAC<br>GACCGAGCGTAGCGAGTCAGTGAGCGAGGAAGCGGAAG
F1 ori (M13 packaging signal)	GCATTAAGCGCGGCGGGTGTGGTGGTTACGCGCAGCGTGACCGCTACACTTGCCAGCGCCCTAGCGCCCGCTCCTTTCGCTTTCTTCCCTTCCTTTCTCGCCA<br>CGTTCGCCGGCTTTCCCCGTCAAGCTCTAAATCGGGGGCTCCCTTTAGGGTTCCGATTTAGTGCTTTACGGCACCTCGACCCCAAAAAACTTGATTAGGGTGA<br>TGGTTCACGTAGTGGGCCATCGCCCTGATAGACGGTTTTTCGCCCTTTGACGTTGGAGTCCACGTTCTTTAATAGTGGACTCTTGTTCCAAACTGGAACAA
terminator B0014	TCACACTGGCTCACCTTCGGGTGGGCCTTTCTGCGTTTATATACTAGAGAGAGAATATAAAAAGCCAGATTATTAATCCGGCTTTTTTATTATTT
**Note: prefix/suffix Csy4 motif RBS Start codon Stop codon Cas9 NGG motif Linker Promoter**	
