# Supplementary material for: Engineering intercellular communication using M13 phagemid and CRISPR-based gene regulation for multicellular computing in Escherichia coli
Source: Nat Commun. 2025 Apr 15;16:3569. doi: 10.1038/s41467-025-58760-z (PMC12000618; doi:10.1038/s41467-025-58760-z)
Supplement: Supplementary file 2 — Reporting Summary [file 41467_2025_58760_MOESM2_ESM.pdf]

nature portfolio

Corresponding author(s): Hadiastri Kusumawardhani & Yolanda Schaerli

Last updated by author(s): Mar 31, 2025

Reporting Summary

Nature Portfolio wishes to improve the reproducibility of the work that we publish. This form provides structure for consistency and transparency in reporting. For further information on Nature Portfolio policies, see our Editorial Policies and the Editorial Policy Checklist.

Statistics

For all statistical analyses, confirm that the following items are present in the figure legend, table legend, main text, or Methods section.

n/a	Confirmed	
☐	☒	The exact sample size (*n*) for each experimental group/condition, given as a discrete number and unit of measurement
☐	☒	A statement on whether measurements were taken from distinct samples or whether the same sample was measured repeatedly
☒	☐	The statistical test(s) used AND whether they are one- or two-sided<br>*Only common tests should be described solely by name; describe more complex techniques in the Methods section.*
☒	☐	A description of all covariates tested
☒	☐	A description of any assumptions or corrections, such as tests of normality and adjustment for multiple comparisons
☐	☒	A full description of the statistical parameters including central tendency (e.g. means) or other basic estimates (e.g. regression coefficient) AND variation (e.g. standard deviation) or associated estimates of uncertainty (e.g. confidence intervals)
☒	☐	For null hypothesis testing, the test statistic (e.g. *F*, *t*, *r*) with confidence intervals, effect sizes, degrees of freedom and *P* value noted<br>*Give P values as exact values whenever suitable.*
☒	☐	For Bayesian analysis, information on the choice of priors and Markov chain Monte Carlo settings
☒	☐	For hierarchical and complex designs, identification of the appropriate level for tests and full reporting of outcomes
☒	☐	Estimates of effect sizes (e.g. Cohen's *d*, Pearson's *r*), indicating how they were calculated

Our web collection on statistics for biologists contains articles on many of the points above.

Software and code

Policy information about availability of computer code

Data collection	Flow cytometry data was collected using NovoExpress software (version 1.6.2).
Data analysis	All data were analysed and visualized using ggplot2 package in R (RStudio 1.4.1106 (R 3.4.0)).

For manuscripts utilizing custom algorithms or software that are central to the research but not yet described in published literature, software must be made available to editors and reviewers. We strongly encourage code deposition in a community repository (e.g. GitHub). See the Nature Portfolio guidelines for submitting code & software for further information.

Data

Policy information about availability of data

All manuscripts must include a data availability statement. This statement should provide the following information, where applicable:

- Accession codes, unique identifiers, or web links for publicly available datasets
- A description of any restrictions on data availability
- For clinical datasets or third party data, please ensure that the statement adheres to our policy

The source data underlying Figs. 2-5 are provided as a Source Data file. The plasmids used in this study (Table S2) and their annotated sequences are available through Addgene [https://www.addgene.org/Yolanda_Schaerli/] Addgene ID #235447-235486.

Research involving human participants, their data, or biological material

Policy information about studies with human participants or human data. See also policy information about sex, gender (identity/presentation), and sexual orientation and race, ethnicity and racism.

Reporting on sex and gender	Not Applicable
Reporting on race, ethnicity, or other socially relevant groupings	Not Applicable
Population characteristics	Not Applicable
Recruitment	Not Applicable
Ethics oversight	Not Applicable

Note that full information on the approval of the study protocol must also be provided in the manuscript.

Field-specific reporting

Please select the one below that is the best fit for your research. If you are not sure, read the appropriate sections before making your selection.

- [x] Life sciences
- [ ] Behavioural & social sciences
- [ ] Ecological, evolutionary & environmental sciences

For a reference copy of the document with all sections, see nature.com/documents/nr-reporting-summary-flat.pdf

Life sciences study design

All studies must disclose on these points even when the disclosure is negative.

Sample size	All experiments were performed with at least three biological replicates. For flow cytometry analysis, we recorded 30,000 events per replicates.
Data exclusions	No data were excluded from analysis
Replication	All experiments were performed with at least three biological replicates
Randomization	Randomization is not applicable in this study
Blinding	Blinding is not applicable in this study

Reporting for specific materials, systems and methods

We require information from authors about some types of materials, experimental systems and methods used in many studies. Here, indicate whether each material, system or method listed is relevant to your study. If you are not sure if a list item applies to your research, read the appropriate section before selecting a response.

Materials & experimental systems

n/a	Involved in the study	
x		Antibodies
x		Eukaryotic cell lines
x		Palaeontology and archaeology
x		Animals and other organisms
x		Clinical data
x		Dual use research of concern
x		Plants

Methods

n/a	Involved in the study	
x		ChIP-seq
	x	Flow cytometry
x		MRI-based neuroimaging

Plants

Seed stocks

Not Applicable

Novel plant genotypes

Not Applicable

Authentication

Not Applicable

Flow Cytometry

Plots

Confirm that:

- [x] The axis labels state the marker and fluorochrome used (e.g. CD4-FITC).
- [x] The axis scales are clearly visible. Include numbers along axes only for bottom left plot of group (a 'group' is an analysis of identical markers).
- [x] All plots are contour plots with outliers or pseudocolor plots.
- [x] A numerical value for number of cells or percentage (with statistics) is provided.

Methodology

Sample preparation

Both sender and receiver cells were inoculated from single colonies (at least three biological replicates for each sample) and grown overnight at 37 C with 1000 rpm shaking in 400 microliter of 2x Yeast Extract Tryptone medium (2x YT) with appropriate antibiotics added. We diluted these overnight cultures ten-fold into fresh 2x YT media (1 mL) and let the cultures grow at 37 C with 1000 rpm shaking for 1 hour (early log phase). Afterwards, absorbance of the cultures was measured and if necessary, a dilution was performed to ensure that all the samples had an OD600 0.5. We mixed sender and receiver cells at 1:1 or 2:1 ratio for experiments with constitutive phagemid production and at a ratio of 3:1 for inducible phagemid production. We added 40 microliter of this mixture, chemical inducer(s) (as indicated), and 360 microliter of 2x YT with kanamycin 50 mg liter-1 (for constitutive phagemid transfer) or 2x YT with kanamycin 50 mg liter-1 and spectinomycin 50 mg liter-1 (for inducible phagemid transfer) into a 2 mL 96x deep well plate. We incubated the deep-well plate at 37 C with 1000 rpm shaking for 4-6 hours. Following the incubation, we diluted the samples 200 times with 1x PBS (pH 7.4) and analysed them using a Novocyte Flow cytometer.

Instrument

Novocyte Flow cytometer

Software

NovoExpress software (version 1.6.2)

Cell population abundance

The relevant cell populations, measured as singlet cell events are present in abundance. Approximately more than 90% of the total measured cell events (after initial gating against particles) are singlet cells.

Gating strategy

First, to discriminate between cells and other particles, all measured events were gated by forward scatter height (FSC.H) > 1000 arbitrary unit (a.u.) and side scatter height (SSC.H) > 200 a.u (gate P1). Second, we excluded doublets by plotting the FSC.H against the forward scatter area (FSC.A) and set a gate for the events with approximately 1:1 ratio of FSC.H to FSC.A (gate E4). We recorded 30,000 events of singlet cells (gate E4). We then set a gate to select for the receiver cells ('sender/receiver threshold' in Figure 2C) for red fluorescence (PE.Texas.Red.H) above 2000 a.u (gate R2). For calculating the fraction of receiver cell population that received the phagemid messages (Figure 2D), we set additional gates for the 'on' state and 'off' sates by setting a threshold for green fluorescence (FITC.H) above or below 10,000 a.u. (gate R3), respectively. For sender cells quantification (Figure 2G), after gating the events with gate P1 and gate E4, we then set a gate to select for the receiver cells by setting thresholds for red fluorescence (PE.Texas.Red.H) above 1000 a.u and green fluorescence (FITC.H) above 1000 a.u. (gate R2). After that, we defined the threshold for receiver cells that received sgRNA-2 (sg2_pos) to be below 30,000 a.u at the green fluorescence (FITC.H) channel, otherwise it is classified as not receiving sgRNA-2 (sg2_neg). Similarly, we defined the threshold for receiver cells that received sgRNA-3 (sg3_pos) to be below 30,000 a.u at the red fluorescence (PE.Texas.Red.H) channel, otherwise it is classified as not receiving sgRNA-3 (sg3_neg). Cell counts for were recorded and fraction of receiver cells with sfGFP repression were reported by dividing sg2_pos cell counts from the total of sg2_pos and sg2_neg cell counts. Gating strategy has been added as Supplementary Figure 12.

- [x] Tick this box to confirm that a figure exemplifying the gating strategy is provided in the Supplementary Information.
